# Mechanistic Study of Glucose Photoreforming over TiO_2_-Based Catalysts for H_2_ Production

**DOI:** 10.1021/acscatal.3c00858

**Published:** 2023-06-13

**Authors:** Lan Lan, Helen Daly, Rehana Sung, Floriana Tuna, Nathan Skillen, Peter K. J. Robertson, Christopher Hardacre, Xiaolei Fan

**Affiliations:** † Department of Chemical Engineering, School of Engineering, The University of Manchester, Manchester M13 9PL, United Kingdom; ‡ Manchester Institute of Biotechnology, The University of Manchester, Manchester M13 9PL, United Kingdom; § Department of Chemistry, University of Manchester, Manchester, M13 9PL, United Kingdom; ∥ Photon Science Institute, University of Manchester, Manchester, M13 9PL, United Kingdom; ⊥ School of Chemistry and Chemical Engineering, Queen’s University Belfast, Belfast BT9 5AG, United Kingdom

**Keywords:** Photoreforming, Hydrogen, Glucose conversion, ATR-IR spectroscopy, Isotopic

## Abstract

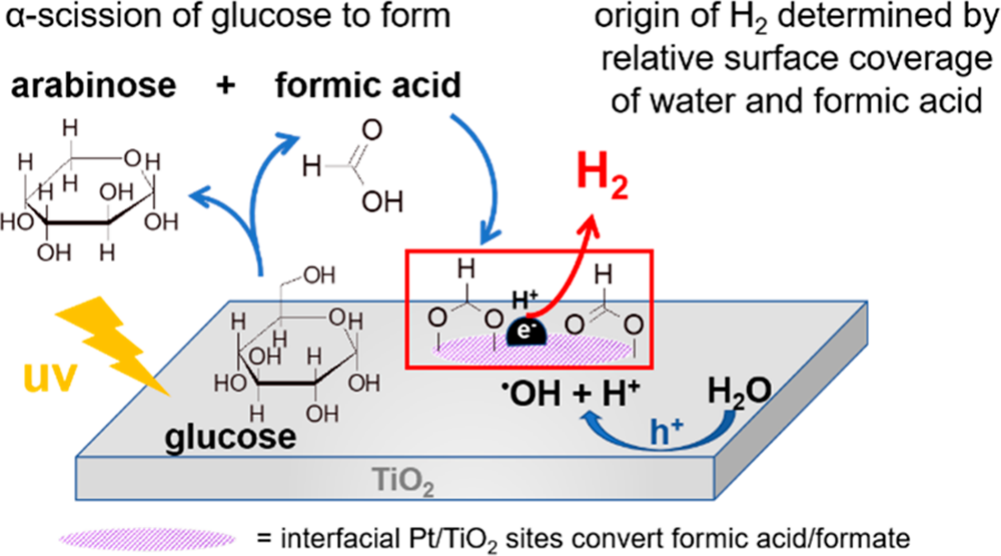

Glucose is a key intermediate in cellulose photoreforming for H_2_ production. This work presents a mechanistic investigation of glucose photoreforming over TiO_2_ and Pt/m-TiO_2_ catalysts. Analysis of the intermediates formed in the process confirmed the α-scission mechanism of glucose oxidation forming arabinose (C_n-1_ sugar) and formic acid in the initial oxidation step. The selectivity to sugar products and formic acid differed over Pt/TiO_2_ and TiO_2_, with Pt/TiO_2_ showing the lower selectivity to formic acid due to enhanced adsorption/conversion of formic acid over Pt/TiO_2_. *In situ* ATR-IR spectroscopy of glucose photoreforming showed the presence of molecular formic acid and formate on the surface of both catalysts at low glucose conversions, suggesting that formic acid oxidation could dominate surface reactions in glucose photoreforming. Further *in situ* ATR-IR of formic acid photoreforming showed Pt-TiO_2_ interfacial sites to be key for formic acid oxidation as TiO_2_ was unable to convert adsorbed formic acid/formate. Isotopic studies of the photoreforming of formic acid in D_2_O (with different concentrations) showed that the source of the protons (to form H_2_ at Pt sites) was determined by the relative surface coverage of adsorbed water and formic acid.

## Introduction

Water splitting is regarded as a sustainable way of producing H_2_ by absorbing and transferring the energy from photons in sunlight and was first reported in 1972 by Fujishima and Honda.^1^ Water dissociation is, however, not thermodynamically favorable, which results in the inefficient production of H_2_ since it is an endergonic transformation (Gibbs free energy, Δ*G*^0^ = +237.1 kJ mol^–1^).^2^ Therefore, strategies have been proposed to address this drawback, such as aqueous phase reforming (APR) of oxygenated organic substances,^3^ which is thermodynamically more favorable than the overall water splitting reaction, e.g., Δ*G*^0^ of methanol photoreforming = +8 kJ mol^–1^,^4^ and Δ*G*^0^ of sucrose photoreforming = −84.7 kJ mol^–1^.^2^ In 1980, Kawai and Sakata first found that in the presence of water and photocatalysts such as RuO_2_/TiO_2_/Pt^5^ and Pt/TiO_2_,^6^ the photocatalytic conversion of sugar, or polysaccharides such as starch and cellulose, led to H_2_ production under the irradiation of a Xe lamp. The H_2_ production rate (*r*_*H*_2__) from photoreforming of sugar, starch and cellulose in aqueous systems (using RuO_2_/TiO_2_/Pt as the catalyst) was reported to be14.0 μmol h^–1^, 10.2 μmol h^–1^ and 3.5 μmol h^–1^, respectively, whereas *r*_*H*_2__ was only 0.2 μmol h^–1^ for pure water splitting.^5^ These studies demonstrated the feasibility of H_2_ evolution from photocatalytic reforming of biomass-derived substrates. Mechanistic understanding of these photoreforming processes (with multiple intermediates/oxidation steps), however, is needed to not only improve H_2_ production but also to realize the potential to produce value-added liquid phase products selectively.

The mechanism of H_2_ production from photocatalytic reforming of cellulose has been investigated from analysis of the liquid/gas products from the reaction^7,8^ and kinetic studies of the reaction.^9,10^ Relevant findings suggested that over the TiO_2_-based catalysts, hydrolysis^7^ or peeling^8^ of cellulose to glucose was the first step, with the glucose proposed to be the main reactant in the liquid phase responsible for H_2_ production in cellulose photoreforming.^9^ In addition, it was suggested that either the holes (h^+^) in the valence band of the catalyst (generated by light irradiation on the catalyst) or ·OH radicals (generated by the reaction of water with the h^+^) were responsible for cellulose depolymerization to glucose which was then proposed to be the primary sacrificial electron donor in the system. The mechanism of glucose formation in cellulose photoreforming, and the mechanism of glucose photoreforming over TiO_2_-based catalysts are, however, currently not well understood.^11−17^

To date, the reaction pathways of glucose photoreforming were predominantly determined from the analysis of gaseous (CO_2_, CO, CH_4_) and liquid products. The glucose conversion pathway over Rh/TiO_2_ was proposed to occur via α-scission (C_1_–C_2_), which was initiated by reactive oxygen species from photo-oxidation of water.^11^ The findings showed that the main products from photoreforming of glucose (C6), arabinose (C5) and erythrose (C4) were C_n-1_ compounds, arabinose, erythrose, and glyceraldehyde (C3), respectively. This indicated that α-scission (in a sequential manner) was common in photocatalytic conversion of sugar aldoses over TiO_2_-based catalysts. Formic acid was produced during the sequential α-scission of the sugar aldose chain together with gaseous products of H_2_ and CO_2_. Based on this mechanism, H_2_ was considered to be produced through dehydrogenation and C–C bond cleavage of glucose.^11,15,16^ Arabinose, erythrose and formic acid were also measured as the main products in the liquid phase of glucose photoreforming over Pt/TiO_2_^16^ and W-/N-doped Pt/TiO_2_ catalysts,^15^ which were proposed to follow the mechanism of C_1_–C_2_ scission and dehydrogenation of glucose. Additionally, α-scission of glucose at the C_1_–C_2_ position was also confirmed by ^13^C NMR analysis, which showed a decrease in the signal of ^13^C labeled glucose in the C_1_ position^18,19^ with formate and carbonate remaining on the TiO_2_ surface after the reaction.^20^ Other studies, however, also reported gluconic acid as the predominant^13^ or only^14^ product from glucose photoreforming over metal (Au, Pd) deposited-TiO_2_ and ZnS/ZnIn_2_S_4_ catalysts. Other mechanisms have also been proposed for glucose photoreforming with initial formation of glucose radicals from interaction of glucose and photogenerated h^+^, which subsequently reacted with other glucose molecules to drive the photoreforming reactions proposed by Fu et al.^21^ Zhou et al.^12^ also proposed a mechanism based on glucose radicals (RC·OH) which were formed initially at the surface of TiO_2_, and then reacted further with water and ·OH to produce CO_2_ via RCOOH.

Oxidation of organic substrates has been proposed to occur through direct (h^+^) and indirect oxidation (·OH) pathways determined by the strength of adsorption of the organic on the surface of the catalyst,^22^ and the substrate to water ratio.^23,24^ Physisorbed species are proposed to undergo indirect oxidation by ·OH radicals (·OH radicals from water oxidation by the photogenerated holes, h^+^) and chemisorbed species, direct oxidation by h^+^ or indirect oxidation by ·OH. As the nature of adsorbed species on the catalyst surface could influence the oxidizing species and reaction pathway (intermediates adsorbed on the catalyst and/or in solution), determination of adsorbed species on the catalyst surface from an aqueous solution (substrate and intermediates), is an important consideration in understanding the photoreforming mechanism.

Attenuated total reflectance infrared (ATR-IR) spectroscopy has been utilized to study the liquid–solid interface to probe adsorption on a catalyst surface (dark adsorption^25,26^) and also under *in situ* conditions with irradiation of UV light.^27−31^ For example, an ATR-IR study of oxalic acid and formic acid adsorption on TiO_2_ (individually and competitively under dark conditions) showed that the oxalic acid-TiO_2_ interaction (via bidentate-chelating) was much stronger than the formic acid-TiO_2_ interaction via bridging adsorption.^25^ Such insight supported the improved H_2_ production from oxalic acid photoreforming (∼57 μmol L^–1^ H_2_) compared to formic acid photoreforming (∼23 μmol L^–1^ H_2_) over TiO_2_, which was attributed to the relatively stronger oxalic acid-TiO_2_ interaction allowing more effective transfer of photogenerated electrons, e^–^ (or h^+^) suppressing the recombination of e^–^-h^+^ pairs.

*In situ* ATR-IR of glyoxylic acid adsorption on a TiO_2_ film under UV irradiation was performed by Ekström et al.^27^ Under UV irradiation, the absorption peaks of adsorbed glyoxylic acid decreased, and peaks of adsorbed oxalate appeared as a function of irradiation time, which clearly confirmed the conversion of the adsorbed glyoxylic acid to the adsorbed oxalate. Similarly, the formation of intermediates (i.e., acetaldehyde and acetic acid) from ethanol photo-oxidation over Pt/TiO_2_ was also observed by ATR-IR spectroscopy under UV irradiation.^32^ Herein, an *in situ* ATR-IR spectroscopy of m-TiO_2_ and Pt/m-TiO_2_ (i.e., mixed phase of 85% anatase and 15% rutile) under UV irradiation was carried out to probe the species adsorbed on the surface of the catalyst during the photoreforming of glucose. This was combined with detailed product analysis of the system (liquid phase and gaseous products via high-performance liquid and gas chromatography, HPLC and GC) to propose a complete reaction mechanism for glucose photoreforming which was confirmed to be via an α-scission mechanism to a C_*n*–1_ compound and formic acid. In addition, isotope mass spectrometry was also employed to study the origin of H_2_ in glucose and formic acid photoreforming over Pt/m-TiO_2_ catalysts.

## Experimental Section

### Chemicals and Materials

The catalyst used in glucose photoreforming was Pt/m-TiO_2_ (i.e., 0.16 wt % Pt loading on a mixture TiO_2_ of 85 wt % anatase and 15 wt % rutile), which was prepared by the impregnation method, and the charaterisation of this catalyst was described previously.^10^

Chemicals used in photoreforming were purchased from Sigma-Aldrich (d-(+)-glucose, dl-glyceraldehyde, glycolic acid, and formic acid), Acros (d-(−)-arabinose), Fisher (formaldehyde, 39 wt %), Glentham life sciences (d-(−)-erythrose), and Fluorochem (Dihydroxyacetone, DHA). Deuterium oxide (D_2_O) water was purchased from Sigma-Aldrich and formic-d acid was purchased from Apollo scientific for the study in isotope mass spectrometry. Sulfuric acid (>98%) used for preparing the mobile phase in HPLC was purchased from Fisher Scientific. 5,5-Dimethyl-1-pyrroline N-oxide (DMPO) used as a spin trap for electron paramagnetic resonance spectroscopy was purchased from Sigma-Aldrich. Deionized (DI) water was obtained from the Direct-Q 3UV ultrapure water system (Millipore).

### Activity of Glucose Photoreforming

The photoreforming of glucose, as well as the identified intermediates (i.e., arabinose, erythrose, glyceraldehyde, and glycolic acid), was performed in a photoreactor (161 mL jacketed borosilicate glass reactor) equipped with a UV-A lamp (peak wavelength of 365 nm, 2 × 8 W bulbs, Thistle Scientific). The photoreactor was connected to a GC (PerkinElmer Clarus 580, fitted with 2 m inline HayeSep DB 100/120 mesh columns followed by a 2-m ShinCarbon ST 100/120 mesh column equipped with TCD and FID detectors) for online analysis of gaseous products. In a typical photocatalytic test, 75 mg of the catalyst was suspended in 100 mL of water (H_2_O or D_2_O) containing 100 mg of substrates. The suspension was mixed and purged with argon for 30 min to remove the dissolved oxygen in water, and then the initial gas sample in the headspace was taken automatically and analyzed by GC. The UV-A lamp was then switched on and 1 mL liquid samples were collected at 30 min intervals during the reaction for 3 h. Gas samples were also analyzed every 30 min. During the photocatalysis, a circulating bath was used to circulate water through the jacket of the reactor to maintain a constant reaction temperature at 40 °C. The dark reactions were performed under the same conditions without light irradiation. Photodecomposition reactions were also carried out under the same conditions but without the addition of the photocatalyst. The concentration of the species in the collected liquid sample was analyzed by HPLC (Agilent 1200 equipped with a refractive index (RI) detector) with an Aminex HPX-87H column operating at 50 °C, and 5 mM H_2_SO_4_ was used as mobile phase at a flow rate of 0.6 mL min^–1^.

For the isotopic study, reactions were performed in D_2_O/H_2_O with glucose or formic acid as the substrate, and in H_2_O with d-formic acid (D-COOH) as the substrate. Gas samples from the headspace of the photoreactor were analyzed by GC (for quantification), and mass spectrometry (Hiden HPR-20 MS) was used to analyze the isotopes in the products (H_2_, D_2_ or HD) after 5 h UV irradiation. Ar (at 40 mL min^–1^) was used as the carrier gas and regulated using Brooks Instruments Mass Flow controllers. Gas samples were delivered by Ar to the MS and analyzed via MASSOFT10 software. An in-line valve with a septum seal was fitted to the Ar line, which enabled the injection of the collected gaseous samples (from the headspace) into the Ar stream. Each time, 70 μL of the gas sample was injected in the MS, and each injection was repeated 6 times. Finally, 70 μL of the final sample (after 5 h photoreforming) was also injected in the GC to obtain the amount of gas samples (i.e., H_2_ and CO_2_). The fragment ions studied were *m*/*z* (mass to charge ratio) = 2, 3, 4, 15, 16, 17, 18, 19, 20, 28, 32, and 44, representing H_2_ (2), HD (3), D_2_ (4), CH_4_ (15 and 16), H_2_O (17 and 18), HDO (17, 18 and 19), D_2_O (18 and 20), N_2_/CO (28), O_2_ (32), and CO_2_ (44 and 28), respectively. The quantification of each mass/fragment was determined by eq 1.
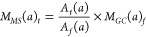
1where *M*_*MS*_ (*a*)_*t*_ represents the amount of gas *a* (μmol) at irradiation time *t*. *A*_*t*_ (*a*) and *A*_*f*_ (*a*) represent the average peak area of gas *a* at irradiation time *t* and in the final sample (at 5 h) obtained from MS, respectively. *M*_*GC*_ (*a*)_*f*_ represents the amount of gas *a* in the final sample measured by GC.

Conversion of the substrate in photoreforming was calculated by eq 2.
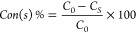
2where *C*_0_ is the initial concentration of the substrate, and *C*_*s*_ is the final concentration of the substrate after reaction.

Selectivity to the liquid phase product was determined by eq 3.
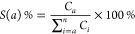
3where *C*_*a*_ is the concentration of a species produced in the liquid phase, and ∑_*i* = *a*_^*n*^*C*_*i*_ is the sum of the concentration of all species produced in the liquid phase.

The photonic efficiency (η) of the system was determined by eq 4.
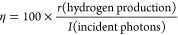
4where *r* (hydrogen production) is the reaction rate for H_2_ formation during the photoreforming in units of μmol h^–1^, and *I* (incident photons) is the incident photon flux in units of Einstein h^–1^ (1 Einstein of photons = 1 mol (6.02 × 10^23^) photons) was titrated by a potassium ferrioxalate actinometre which was described by Bolten et al. elsewhere.^33^

### *In Situ* ATR-IR Spectroscopy under UV Irradiation

*In situ* ATR-IR spectra were recorded using a PIKE ATRMaxII accessory with an in-house built ATR cell (as shown in Figure S1, Electronic Supporting Information, ESI) housed in a Bruker Tensor II spectrometer. The ATR cell was composed of three stainless steel plates, which included a baseplate to support the germanium (Ge) ATR crystal, a middle plate with an aperture creating the channel for the liquid/gas flow, as well as hosting a thermocouple to monitor the temperature during photoreforming under UV irradiation. The top plate contained a borosilicate glass window which allowed UV irradiation of the catalyst/substrate solution on the crystal. The light source used in the ATR experiments was UV-LED (emission at 391 nm as detailed in the ESI), which was placed into the aperture of the top plate of the cell to illuminate the catalyst layer coated on the Ge crystal.

The supported catalyst layer (m-TiO_2_ or 0.16 wt % Pt/m-TiO_2_) was prepared by dropping 1 mL of the catalyst slurry (0.1 mg catalyst in 20 mL DI water) onto the surface of the Ge crystal, and the layer was dried via evaporation at room temperature overnight. Before ATR experiments, water and solutions were purged with pure nitrogen for 10 min to remove dissolved oxygen.

In a typical experiment, the background spectrum of the dry catalyst layer was recorded prior to the introduction of water or the substrate solution (0.1 mol L^–1^) to the cell. ATR spectra of the catalyst layer under the water/substrate solution were recorded and then excess water/substrate solution was purged from the cell to leave a water/substrate/catalyst layer (bands due to liquid water were observed throughout the experiment under UV irradiation and these bands were subtracted from all spectra). The head space above the solution/catalyst film was then flushed with N_2_ to ensure an anaerobic environment and sealed before illumination.

## Results and Discussion

### Gas and Liquid Products Analysis for Glucose Photoreforming

**Figure 1:**
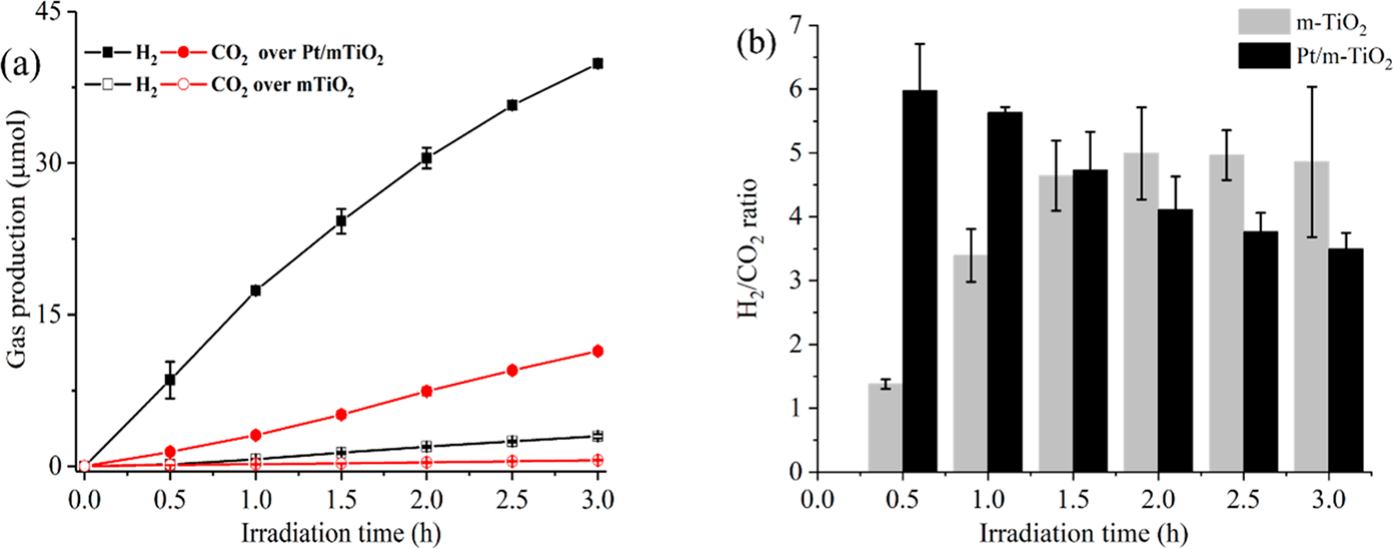
(a) Time course of H_2_ (black) and CO_2_ (red) production over Pt/m-TiO_2_ (solid symbols) and m-TiO_2_ (hollow symbols). (b) The ratio of produced H_2_/CO_2_ as a function of irradiation time in glucose photoreforming. Reaction conditions: 75 mg of catalyst, 0.006 mol L^–1^ substrate in 100 mL of H_2_O, under the irradiation of UV-A lamp for 3 h at 40 °C. Incident photon rate: 62.9 μmol h^–1^ photons, 1 mol (6.0223 × 10^23^) is 1 Einstein of photons.

Figure 1a shows the gas production (H_2_ and CO_2_) from glucose photoreforming over m-TiO_2_ and Pt/m-TiO_2_ as a function of irradiation time. H_2_ and CO_2_ were produced gradually under UV irradiation from the two systems while no obvious H_2_ production from water splitting (without glucose) was observed. Pt/m-TiO_2_ was more effective than m-TiO_2_ for promoting H_2_ production during glucose photoreforming, i.e., 39.8 vs 3.0 μmol after 3 h reaction. According to eq 4, the photonic efficiency, η was calculated as 21.1% for Pt/m-TiO_2_ and 1.6% for m-TiO_2_, respectively, showing that Pt nanoparticles enhanced the separation of photogenerated e^–^ and h^+^ in m-TiO_2_,^9^ which benefited H_2_ production from the photoreforming system with Pt/m-TiO_2_. Comparatively, CO_2_ production from the photoreforming systems was low possibly due to intermediate production via the oxidation pathway. Initially, the ratio of H_2_/CO_2_ production (as shown in Figure 1b below) is much higher over Pt/m-TiO_2_ (around 6) than that over m-TiO_2_ (around 1.4). The ratio then decreased over Pt/m-TiO_2_, while the opposite was found for the system employing m-TiO_2_ under UV irradiation. After 3 h, the ratio of H_2_:CO_2_ was 3.5 and 4.9 for Pt/m-TiO_2_ and m-TiO_2_, respectively, which was higher than that of the theoretical stoichiometric value of glucose reforming in water (i.e., 2:1). This suggests that, under these reaction conditions, (i) protons were derived predominantly from water and (ii) intermediate oxidation products were formed in the liquid phase. The decreasing H_2_/CO_2_ ratio over Pt/m-TiO_2_ also indicates conversion of the intermediates (to CO_2_) during the reaction. Conversely, over m-TiO_2_, the increase and stabilization of the H_2_/CO_2_ ratio suggests m-TiO_2_ is less active for the conversion of intermediates formed in glucose photoreforming.

**Figure 2:**
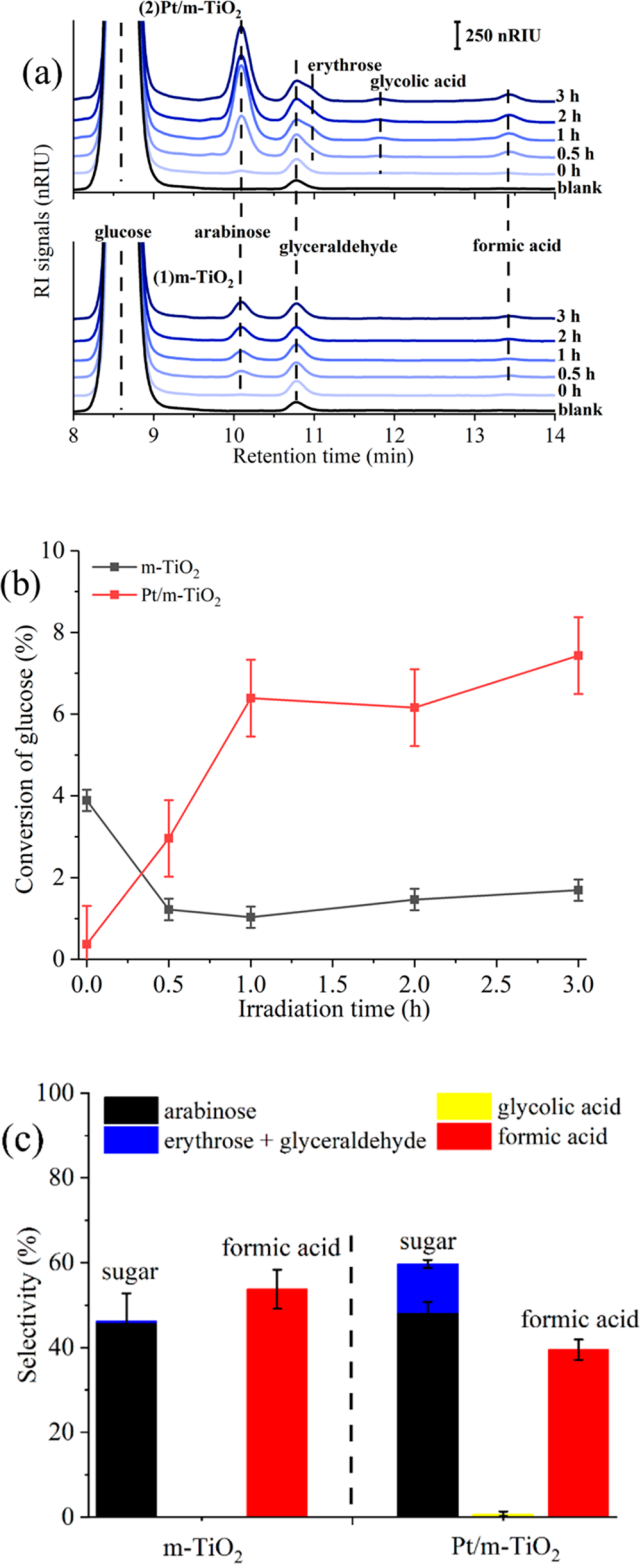
(a) HPLC analysis of product distribution in glucose photoreforming over (1) m-TiO_2_ and (2) Pt/m-TiO_2_; (b) glucose conversion in photoreforming over m-TiO_2_ (black line) and Pt/m-TiO_2_ (red line); (c) averaged selectivity to liquid-phase products in glucose photoreforming (0.5–3 h in Figure S3) over m-TiO_2_ and Pt/m-TiO_2_. Reaction conditions: 75 mg of catalyst, 0.006 mol L^–1^ substrate in 100 mL of H_2_O, under the irradiation of the UV-A lamp for 3 h at 40 °C.

The product distribution in the liquid phase from the m-TiO_2_ and Pt/m-TiO_2_ systems was analyzed as a function of irradiation time and shown in Figure 2a. With m-TiO_2_ (as shown in Figure 2a-(1)), arabinose, glyceraldehyde and formic acid were measured after 30 min irradiation, and their peak intensity increased gradually during photoreforming. In the system over Pt/m-TiO_2_ (as shown in Figure 2a-(2)), arabinose, glyceraldehyde, and formic acid were measured as well, however, at higher concentrations compared to the m-TiO_2_ system. Additionally, traces of erythrose (i.e., the coeluting peak next to the glyceraldehyde peak, as a shoulder at a higher retention time) and glycolic acid were also observed to accumulate in the liquid phase for the Pt/m-TiO_2_ system (Figure 2a). The findings show that the extent of glucose oxidation over the Pt catalyst was more significant than that over pristine m-TiO_2_.

Adsorption of glucose on the catalysts was observed at the initial stage of the reaction (at 0 h) in the dark,^19^ i.e., ∼3.9% over m-TiO_2_ and ∼1.0% over Pt/m-TiO_2_, respectively, as shown in Figure 2b, and neither intermediates/products in the liquid phase (Figure 2a) nor gas phase (Figure 1a) were observed. Under UV irradiation, glucose conversion over m-TiO_2_ was insignificant at 1.2 ± 0.3%. Conversely, the conversion increased initially (<1 h) with the presence of Pt/m-TiO_2_, then leveled off at 7.4 ± 0.9% after 1 h irradiation.

Figure S3 (ESI) shows the selectivity to the products identified by HPLC as a function of irradiation time. The selectivity to arabinose (45–52% over Pt/m-TiO_2_, 40–56% over m-TiO_2_) and formic acid (37–43% over Pt/m-TiO_2_, 50–61% over m-TiO_2_) was high in both systems, while erythrose and glycolic acid were only observed in the Pt/m-TiO_2_ system and with relatively low selectivity. The averaged product selectivity in the two systems during 3 h of reaction was compared (Figure 2c) to investigate the extent of reaction along the glucose oxidation pathway over the two catalysts. The averaged selectivity to arabinose was ∼48.2% over Pt/m-TiO_2_ and ∼45.9% over m-TiO_2_, while the value of formic acid was ∼39.5% over Pt/m-TiO_2_ and ∼53.8% over m-TiO_2_, respectively (Figure 2c). The high selectivity to formic acid and arabinose suggests the α-scission (or C_1_–C_2_ bond cleavage) mechanism for glucose photoreforming.^11,15,16,20^ The averaged selectivity to sugars (arabinose + erythrose) and formic acid showed different phenomena over m-TiO_2_ and Pt/m-TiO_2_. By considering the errors, the averaged selectivity to sugars and formic acid is similar over m-TiO_2_, while the averaged selectivity to formic acid is much less than that of sugars over Pt/m-TiO_2_. According to the α-scission mechanism, formic acid and arabinose (and formic acid and erythrose from arabinose) should be formed in equimolar quantities, which was observed for the m-TiO_2_ system (within error). For Pt/m-TiO_2_, however, the selectivity to total sugars was ∼60% and to formic acid was 40% (Figure 2c). The selectivity was calculated based on HPLC analysis of the products in the liquid phase, hence, the difference in the selectivity reflects preferential adsorption/conversion of formic acid over Pt/m-TiO_2_ compared to m-TiO_2_.

As the intermediates (arabinose, erythrose, glyceraldehyde, and glycolic acid) were observed over Pt/m-TiO_2_ after only 0.5 h of irradiation of UV irradiation, investigation of photoreforming of relevant individual intermediates was undertaken to gain insight into the complete oxidation pathway in glucose photoreforming.

### Photoreforming of Intermediates

**Figure 3:**
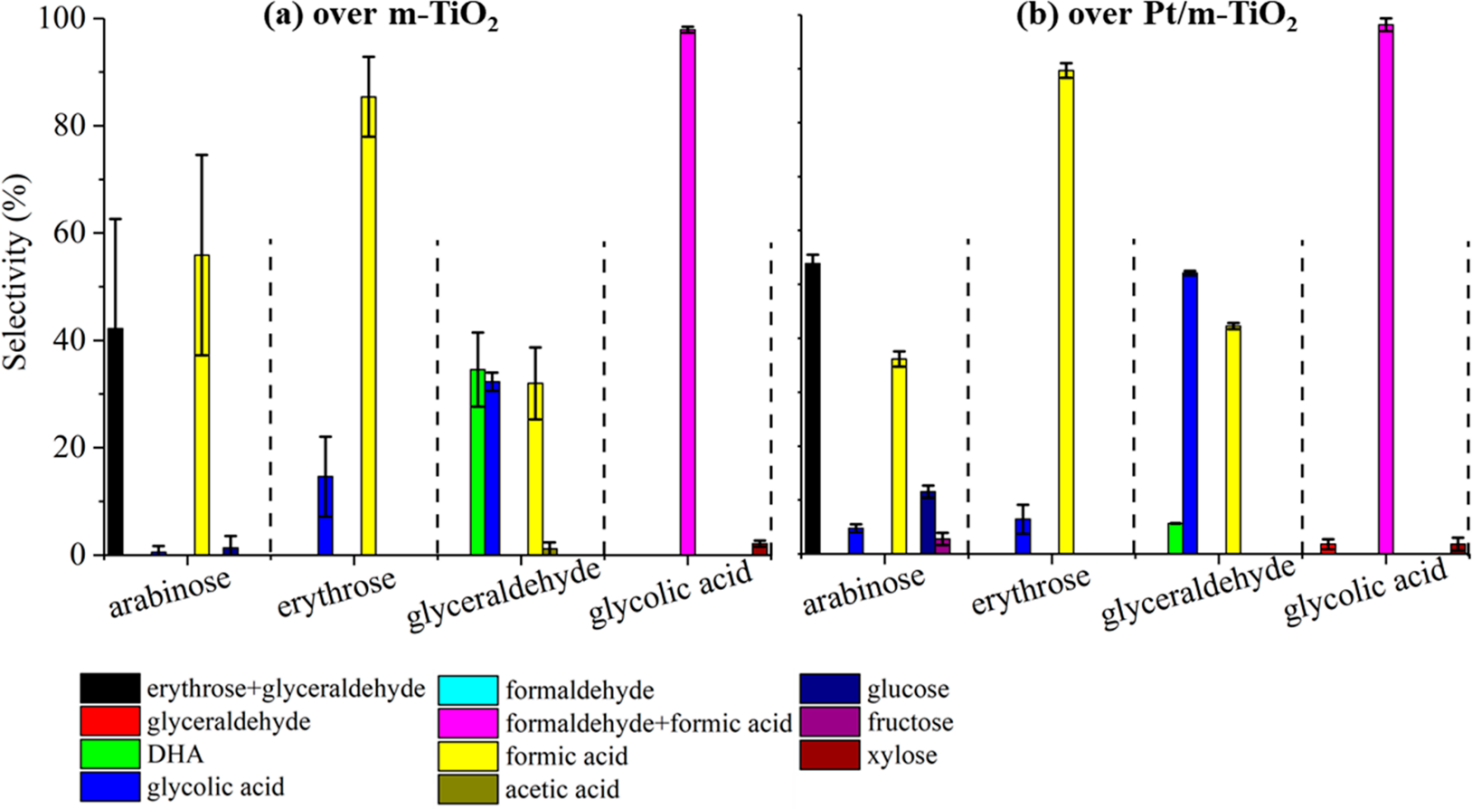
Averaged product selectivity in photoreforming of arabinose, erythrose, glyceraldehyde, and glycolic acid over (a) m-TiO_2_ and (b) Pt/m-TiO_2_. Reaction conditions: 75 mg of catalyst, 0.006 mol L^–1^ substrate in 100 mL of H_2_O, under the irradiation of UV-A lamp for 3 h at 40 °C.

Photoreforming of the intermediates identified from glucose photoreforming was performed to probe the oxidation rate and product selectivity. The selectivity to different products in these photoreforming reactions did not change significantly under UV irradiation, especially after 1 h of reaction, as shown in Figure S4 (ESI). Accordingly, averaged selectivities to relevant products in the photoreforming reactions were compared (Figure 3) to study the reaction pathways of the m-TiO_2_ and Pt/m-TiO_2_ system.

In arabinose photoreforming over m-TiO_2_ and Pt/m-TiO_2_, formic acid and erythrose+glyceraldehyde were identified as the main products. The selectivity to the sugar and acid products, however, was different over the two catalysts. Specifically, selectivities of 42.2% to erythrose+glyceraldehyde and 55.9% to formic acid were observed over m-TiO_2_, while selectivities of 53.9% to erythrose+glyceraldehyde and 36.2% to formic acid over Pt/m-TiO_2_. The selectivities agree well with the findings from glucose photoreforming, i.e., m-TiO_2_ was more selective to produce formic acid in the liquid phase, while Pt/m-TiO_2_ was prone to produce sugars (i.e., arabinose and erythrose). The difference in the selectivities of the sugars and formic acid in the liquid phase reflects a difference in the adsorption or oxidation ability of m-TiO_2_ and Pt/m-TiO_2_ for these substrates. For example, the lower selectivity to formic acid over Pt/TiO_2_ suggests preferential adsorption and/or oxidation of formic acid compared to the sugars.

Glycolic acid and formic acid were identified from the two photoreforming systems using erythrose (the product intermediate from arabinose photoreforming) as the substrate, with formic acid as the major product. Glyceraldehyde, however, could not be separated from erythrose using the current HPLC system but was identified as a product from the photoreforming of glucose (Figure 2a) and arabinose (Figure S4a, ESI). Previous studies also suggested glyceraldehyde as the main product from erythrose photoreforming.^11,34^ Therefore, to reveal the complete reaction pathway, glyceraldehyde photoreforming was also carried out over m-TiO_2_ and Pt/m-TiO_2_. As shown in Figure 3, dihydroxyacetone (DHA) was formed in glyceraldehyde photoreforming over m-TiO_2_ and Pt/m-TiO_2_ due to glyceraldehyde isomerization, which was proposed to occur via enediol (formed by hydrogen migration).^35^ The selectivity to DHA was much higher in the m-TiO_2_ system (∼34.5%) than that over the Pt/m-TiO_2_ system (∼5.7%), suggesting that Pt loading alters the reactions pathways with glyceraldehyde isomerization favored over TiO_2_. In addition to glyceraldehyde isomerization (to yield DHA), glyceraldehyde photoreforming under UV irradiation also produced glycolic acid and formic acid (selectivity: 52.1% and 42.3% in the Pt/m-TiO_2_ system vs 32.3% and 32.0% in the m-TiO_2_ system). Glycolic acid and formic acid were measured as well in erythrose photoreforming, which agrees well with the hypothesis of glyceraldehyde formation in erythrose photoreforming.

Glycolic acid was subsequently used as the substrate in the photoreforming systems. In both systems, formaldehyde and formic acid were measured as the main product from glycolic acid photoreforming, i.e., total selectivity of 98.2% over m-TiO_2_ and 97.9% over Pt/m-TiO_2_ (Figure 3). Using the current HPLC method and column, baseline resolution of the peaks of formaldehyde and formic acid was, however, not possible. Figure S5 (ESI) shows that formic acid was identified as a shoulder peak at higher retention time relative to formaldehyde, therefore, the total selectivity of formic acid + formaldehyde was presented with formaldehyde as the majority. Additionally, Figure S5 shows that Pt/m-TiO_2_ was more effective in promoting the formation of formic acid and formaldehyde than m-TiO_2_.

**Scheme 1:**
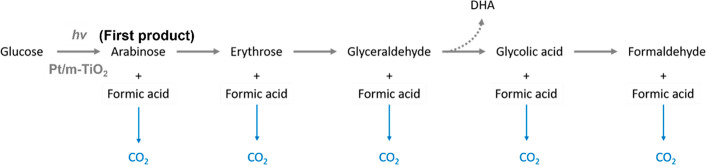
Reaction Pathway for Glucose Oxidation under Photoreforming Conditions over Pt/m-TiO_2_

Based on the analysis and discussion above, the reaction pathway of glucose photoreforming is proposed (Scheme 1). Over Pt/m-TiO_2_, glucose was oxidized via α-scission to produce erythrose, glyceraldehyde, and glycolic acid along with formic acid at each oxidation step. In the photoreforming of glucose over Pt/m-TiO_2_, the amount of H_2_ produced was 39.8 μmol (3 h of reaction). According to the stoichiometry of full glucose photoreforming (C_6_H_12_O_6_ + 6 H_2_O → 12 H_2_ + 6 CO_2_), the theoretical CO_2_ yield would be 19.9 μmol. Only ∼11.4 μmol CO_2_ was produced from the Pt/m-TiO_2_ system, however, because glucose α-scission proceeds with retention of C atoms in the liquid phase intermediates, i.e., C_6_ glucose forms C_5_ arabinose and C_1_ formic acid, and only formic acid could be oxidized to CO_2_.

The findings from the photoreforming systems are in line with the previous studies, which suggest α-scission (C_1_–C_2_) of sugars (to form a C_*n*–1_ sugar and formic acid) as the reaction mechanism in glucose photoreforming.^11,15^ Interestingly, the Pt/m-TiO_2_ system showed the lower selectivity to formic acid in the liquid phase compared to glucose and arabinose, which could be attributed to the favored adsorption/decomposition of formic acid on the surface of Pt/m-TiO_2_. To understand the adsorption behaviors of the intermediates/reactants on the catalyst surface under UV irradiation, *in situ* ATR-IR study of glucose photoreforming was subsequently undertaken.

### *In Situ* ATR-IR Study of Photoreforming under UV Irradiation

ATR-IR spectra of glucose adsorbed over m-TiO_2_ and Pt/m-TiO_2_ are shown in Figure S6. Glucose in water at room temperature exists predominantly as the pyranose ring with a ∼2:1 ratio of the β-anomer to α-anomer with the linear aldose form accounting for only 0.0026 mol %.^36^ Bands in the spectra of 0.1 mol L^–1^ (M) glucose/water were found at 1151, 1109, 1081, 1036, 1018, and 993 cm^–1^ corresponding to the coupled vibrations of ν(C–O) and ν(C–C) stretching, as well as δ(OH) vibrations of glucose (pyranose ring form).^37−40^

The adsorption of glucose over TiO_2_ surfaces has been suggested to occur both dissociatively^41^ and molecularly, and DFT calculations suggest the molecular adsorption of glucose over anatase to be preferred over dissociative adsorption.^42^ Herein, no significant change in the spectra were observed between solutions of 0.1 M glucose/water over the blank Ge crystal (Figure S6a, blue spectrum) and the m-TiO_2_ (Figure S6a, red spectrum) or Pt/m-TiO_2_ (Figure S6a, black spectrum) layers in the dark which could be assigned to molecular, H-bonded interaction of glucose (pyranose ring) over TiO_2_. No bands due to the linear aldose form of the sugar in the dark adsorption were observed (∼1700 cm^–1^ region). Zhou et al. have studied the photoreforming of α-d-glucose, β-d-glucose and the equilibrium mixture of glucose anomers.^12^ The findings showed molecular adsorption of glucose on Pt/TiO_2_ with enhanced H_2_ production from the α-d-glucose epimer, which was attributed to the increased adsorption on the catalyst and more effective trapping of ·OH radicals than that from β-d-glucose.

**Figure 4:**
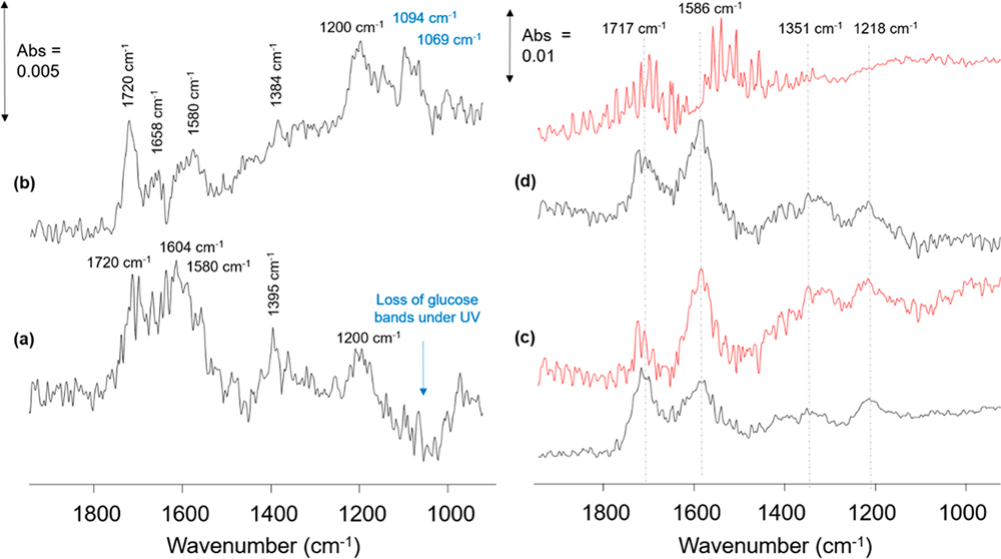
ATR-IR spectra of 0.1 M glucose in water over (a) m-TiO_2_ and (b) 0.16%Pt/m-TiO_2_ after 30 min of UV irradiation and ATR-IR spectra of 0.1 M formic acid in water over (c) m-TiO_2_ and (d) Pt/m-TiO_2_; black spectra: dark adsorption and red spectra: after 60 min of irradiation (LED emissions at 391 nm). Bands due to water have been subtracted from all spectra. The increasing background observed under UV irradiation over Pt/m-TiO_2_ in glucose photoreforming and both catalysts for formic acid photoreforming is a result of IR absorbance by shallow trapped photogenerated electrons.

*In situ* ATR spectra of glucose photoreforming was studied over m-TiO_2_ and Pt/m-TiO_2_ to probe species adsorbed on the catalysts under UV irradiation for the comparison with the liquid phase products observed in the activity tests. The spectra of glucose adsorbed over m-TiO_2_ and Pt/m-TiO_2_ under UV irradiation is shown in Figure 4a,b, as difference spectra where the catalyst-glucose-water spectrum under dark conditions was subtracted from all spectra under UV irradiation. Positive bands in the spectra indicate species formation, and negative bands indicate loss of species adsorbed on the catalyst. Spectra at varied time intervals under UV irradiation are shown in Figure S7 for both m-TiO_2_ and Pt/m-TiO_2_.

On m-TiO_2_, a loss of bands in the 1200–900 cm^–1^ region (i.e., C–C/C-O vibrations of glucose) was observed under UV irradiation (Figure 4a and Figure S7), indicating the removal or degradation of adsorbed glucose under UV light. This was also noted as conversion of glucose was 3.9% in dark (from adsorption) but only 1% under UV irradiation (Figure 2b). In addition, new bands were observed to form at 1720, 1604, (with a shoulder at 1580 cm^–1^), 1395 and 1200 cm^–1^, and these bands were assigned to molecularly and dissociatively adsorbed formic acid (Table 1).^43−53^ The bands at 1720 and 1200 cm^–1^ are assigned to ν(C=O) and ν(C–O) vibrations of formic acid hydrogen bonded to surface hydroxyl groups/adsorbed water with bands due to formates (from the dissociative adsorption of formic acid) observed at 1604 and 1580 cm^–1^ due to ν_*as*_(OCO) while the band at 1392 cm^–1^ could be assigned to ν_*s*_(OCO) or δ(C–H) of adsorbed formate species.^46^ As bands in the 1300–1400 cm^–1^ region are relatively weak, further assignment and identification of the mode of formate adsorption (monodentate vs bridging bidentate) is not performed herein.

**Table 1:** Assignment of Band Positions (cm^–1^) of Species Adsorbed on m-TiO_2_ and 0.16% Pt/m-TiO_2_ Following Glucose and Formic Acid Photoreforming

Glucose		Formic acid	Assignment
TiO_2_	0.16%Pt/TiO_2_	TiO_2_/0.16%Pt/TiO_2_	
1720	1720	1717	*v*(C=O) - molecular (physiorbed) formic acid
	1658		*v*(C=O) - molecular (physiorbed) formic acid
1604	1604 (sh)		*v*_as_(CO_2_^–^) - formate
1580 (sh)	1580	1586	*v*_as_(CO_2_^–^) - formate
1395	1384	1351	*v*_s_(CO_2_^–^)/δ(C–H) - formate
1200	1200	1218	*v*(C–O) - molecular (physiorbed) formic acid
	1094		*v*(C–O), *v*(C–C), β(C–OH) - arabinose
	1069		

Formic acid was observed as a liquid phase product from glucose photoreforming over m-TiO_2_ and showed that comparable reaction products between the two photoreforming systems were observed (i.e., ATR cell under the irradiation of UV-LEDs probing the catalyst-water interface and photoreactor under the irradiation of UV-A lamp). Over m-TiO_2_, bands due to formic acid and formate were observed upon initial exposure of glucose-m-TiO_2_ to UV light with no obvious changes in the band intensity of molecular formic acid or formate species adsorbed on m-TiO_2_ under UV irradiation. This suggested the low activity of m-TiO_2_ for converting formic acid/formate under these conditions.

The *in situ* IR spectrum after 30 min of irradiation over Pt/m-TiO_2_ is shown in Figure 4b. Over Pt/m-TiO_2_, loss of bands due to glucose in the 1200–900 cm^–1^ region was not as pronounced as seen over m-TiO_2_, due to new bands forming in this region at 1094 and 1069 cm^–1^. These bands have been assigned to arabinose according to Figure S6. Over Pt/m-TiO_2_, bands due to molecularly adsorbed formic acid were the predominant species observed under UV irradiation with bands at 1720 and 1200 cm^–1^*ν(*(C=O) and ν(C–O)) due to hydrogen bonded monomeric species and 1658 cm^–1^ to ν(C=O) vibration of an adsorbed dimeric formic acid species.^43^ Significantly weaker bands related to formate species were identified on Pt/m-TiO_2_ under UV irradiation, being different from m-TiO_2_, on which formate species were relatively more intense than molecularly adsorbed formic acid during glucose photoreforming.

The formation of arabinose and formic acid corresponds with the photoreforming activity data and indicates that glucose decomposition occurs via the α-scission mechanism, that is, glucose is initially converted to the C_n-1_ sugar (i.e., arabinose) and formic acid. This is proposed to occur from an initially H-bonded (molecular) adsorption of glucose on the m-TiO_2_ and Pt/m-TiO_2_ catalysts (and attack by ·OH generated by oxidation of water by photogenerated holes).

The differing relative intensity of the formate and molecularly adsorbed formic acid bands observed over m-TiO_2_ and Pt/m-TiO_2_ in glucose photoreforming (under UV irradiation) could, however, suggest the different reactivity of the molecular and dissociatively adsorbed formic acid. To probe this, the photoreforming of formic acid was also studied by ATR-IR over m-TiO_2_ and Pt/m-TiO_2_. Dark adsorption of formic acid over m-TiO_2_ and Pt/m-TiO_2_ is shown in Figure 4 (black spectra) where molecular formic acid (1717 and 1218 cm^–1^) and formate species (1586 and 1350 cm^–1^) were observed over both catalysts (Table 1). Difference spectra for the photoreforming of formic acid over m-TiO_2_ (Figure 4c) showed no changes in the species adsorbed on TiO_2_ under UV irradiation (red spectra). UV irradiation did not result in the conversion of formic acid/formate adsorbed on m-TiO_2_ under these reaction conditions (Figure 4c). The photocatalytic decomposition of formic acid, under anaerobic conditions, utilizes lattice oxygen to form CO_2_ (and H_2_O) and deactivation over TiO_2_ has been linked to the removal of lattice oxygen with water-UV conditions unable to replenish the lattice oxygen (requires gas phase oxygen or slow diffusion of oxygen from the TiO_2_ bulk).^54^ The initial formation of formic acid/formate on m-TiO_2_, herein, could be the result of the reaction with TiO_2_ lattice oxygen which cannot be replenished resulting in deactivation for the conversion of formic acid/formate. In contrast, for Pt/m-TiO_2_, bands due to formates and molecular formic acid disappeared during 30 min of UV irradiation which confirms that Pt sites (Pt–Ti interface and/or Pt sites) are important for photocatalytic decomposition of formic acid (Figure 4d).

For m-TiO_2_, when formic acid was adsorbed as the substrate or formed during glucose photoreforming, no difference was observed in the spectra, with molecular formic acid and formates present which were not degraded under UV irradiation. Over Pt/m-TiO_2_, adsorption of formic acid as the substrate (Figure S8, red spectrum, 0.1 M formic acid, ESI), resulted in adsorption of formates and molecular formic acid on the catalyst. In photoreforming of glucose, however, molecular formic acid was the predominant species on the Pt/m-TiO_2_ with significantly weaker bands due to formate species (Figure S8, black spectrum). The relatively lower intensity of the formate relative to the molecular formic acid bands from glucose photoreforming on Pt/m-TiO_2_ suggests formate conversion (to CO_2_).

The coexistence of both molecular and dissociative formic acid has been proposed to be favorable for the photocatalytic degradation of formic acid.^55^ Over the surface of TiO_2_ (P25^43^ and anatase^56^), formic acid could adsorb both molecularly, and dissociatively forming monodentate and bidentate formates. FTIR spectroscopy and temperature-programmed desorption analyses showed the mode of formate adsorption to change with the addition of water, and different formate adsorption structures to have different reactivities.^57^ Formates were also identified as the active species in the photooxidation/decomposition of formic acid by Turki et al.^55^ with molecular formic acid proposed to resupply the reactive formates.^58^ Here, variation in relative intensity between molecular formic acid and formates observed from glucose photoreforming also indicates that molecular formic acid may be a pool for formates which can be oxidized to CO_2_.

**Scheme 2:**
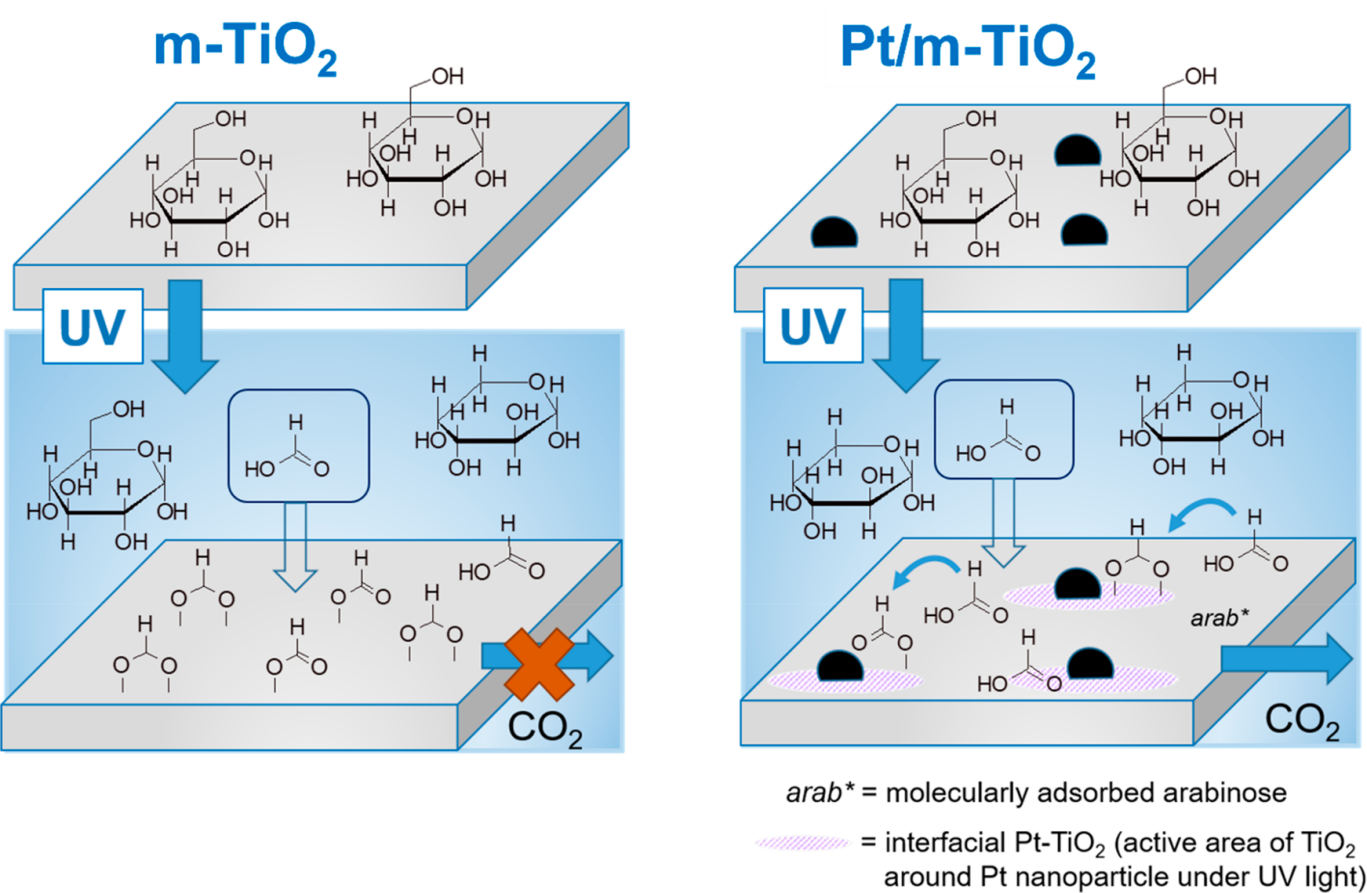
Schematic of Glucose Photoreforming Mechanism over m-TiO_2_ and Pt/m-TiO_2_ with Preferential Adsorption of Formic Acid over Glucose and Conversion of Formates/Formic Acid at Pt–Ti Interface Sites

The ATR-IR study of glucose photoreforming has shown that under UV irradiation, species due to molecular and chemisorbed formic acid were adsorbed on the surface of the catalyst. The adsorption of formic acid, an intermediate product, in the presence of a relatively higher concentration of the substrate glucose, suggests competitive adsorption occurs between the substrate and intermediates, which could determine the oxidation pathway/rate in the overall photoreforming reaction. The differing reactivity of formic acid/formate on m-TiO_2_ and Pt/m-TiO_2_ suggests Pt-TiO_2_ interface sites to be active for formic acid oxidation. An active interfacial area around Pt particles was proposed as well in methanol photoreforming over varied Pt loading Pt/TiO_2_ catalysts.^59^ In glucose photoreforming, initial α-scission of molecularly adsorbed glucose is proposed to occur through •OH attack forming adsorbed formic acid/formate which can undergo oxidation to CO_2_ at Pt-TiO_2_ interfacial sites (Scheme 2).

### Origin of H_2_ in Glucose and Formic Acid Photoreforming

H_2_ and formic acid were produced in glucose photoreforming, as well as the photoreforming of all relevant intermediates (H_2_ production from intermediates is shown in Figure S9, ESI) in the liquid phase. Previously, mechanistic studies of organics photooxidation (such as methanol^60^) showed that formic acid/formate are the terminal intermediates before CO_2_ formation. Formate/formic acid herein, were, however, formed continually during glucose photoreforming being present on the catalyst surface at the initial stage of the reaction (low conversion of glucose) and not just as the final intermediate before CO_2_ formation.

Studies of glucose photoreforming have proposed the production of H_2_ to be from coupling of H^+^ derived from glucose,^12,61^ or water,^12,14,61^ but the presence of formic acid (and sugars/intermediates) on the catalyst surface, offers additional species which could be (preferentially) reformed, driving the photoreforming reaction (enabling charge separation, reaction with holes/·OH species). For formic acid, in addition to photodecomposition/photoreforming, H_2_ could also be produced from direct decomposition (dehydrogenation) under light irradiation^62,63^ or in the dark,^64,65^ and therefore, it is necessary to investigate the mechanism of H_2_ production from formic acid under the conditions of glucose photoreforming.

Catalytic decomposition and photolysis (under UV light without a catalyst) of formic acid were carried out to investigate their possible contributions to H_2_ production in this system. H_2_ produced from the systems without light irradiation and without catalyst were compared to that from the photoreforming reaction (as shown in Figure S10, ESI). From the results, H_2_ produced from the catalytic conversion (i.e., 0.8 μmol at 3 h) or photolysis (i.e., 1.2 μmol at 3 h) of formic acid was far lower than that from formic acid photoreforming (151.9 μmol at 3 h). Therefore, the contribution to the H_2_ production from catalysis or photolysis of formic acid was very insignificant in this system.

**Figure 5:**
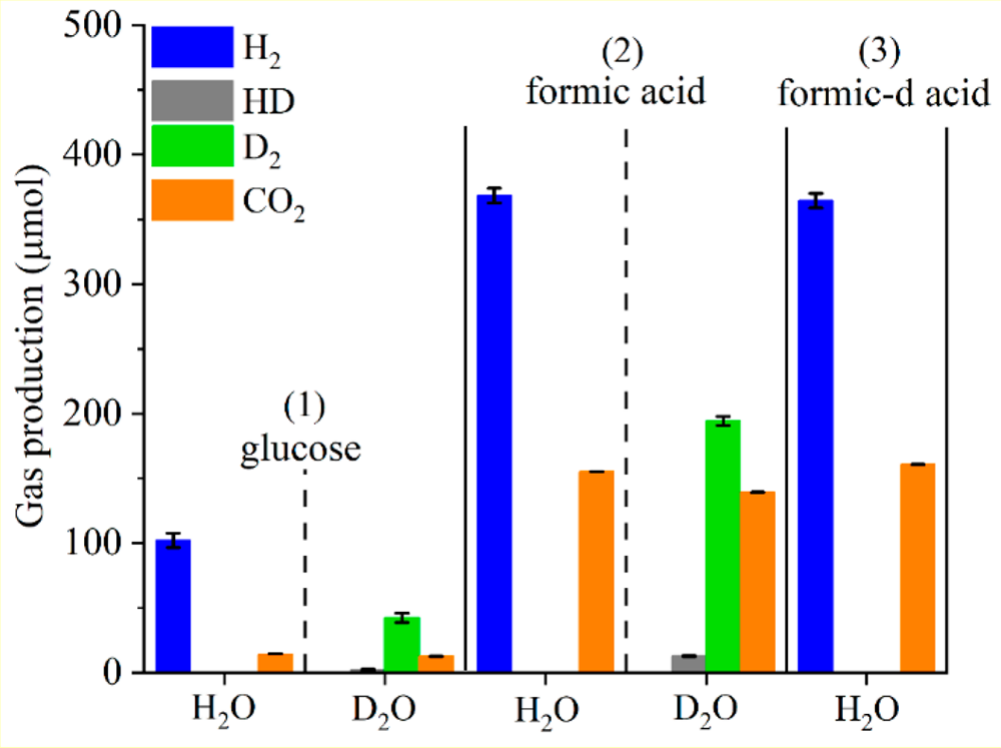
Gas productions (H_2_ and CO_2_) after 5 h irradiation over Pt/m-TiO_2_ of (1) glucose (2) formic acid and (3) formic-d acid photoreforming. Reaction conditions: 75 mg of Pt/m-TiO_2_, 0.006 mol L^–1^ substrate in 100 mL of H_2_O or D_2_O, under the irradiation of UV-A lamp for 5 h at 40 °C.

To probe the mechanism of the H^+^ reduced to H_2_ in photoreforming of glucose and formic acid, isotopic studies were performed in H_2_O or D_2_O over Pt/m-TiO_2_ (Figure S11 in ESI, and Figure 5) using mass spectrometry (MS) to analyze the composition of the gas products. Hydrogen (i.e., H_2_, HD or D_2_) was observed, accumulating gradually under the irradiation of UV-A lamp in photoreforming of glucose (Figure S11a) and formic acid (Figure S11b) in H_2_O or D_2_O. After 5 h irradiation, the amount of hydrogen (H_2_, HD, and D_2_) and CO_2_ produced from the photocatalytic reactions in H_2_O/D_2_O was compared between different systems (as shown in Figure 5).

In the system of glucose in H_2_O, the amount of produced H_2_ was 102.2 μmol, while in the system of glucose in D_2_O, the products were D_2_ (42.3 μmol) with no H_2_ formed and a trace amount of HD (2.3 μmol) which was attributed to OH/OD exchange between glucose and D_2_O (Figure 5-(1)). The results indicate that hydrogen produced in glucose photoreforming is mainly from water (reaction of water with photogenerated holes) and the lower yield of hydrogen produced from reaction in D_2_O compared to H_2_O indicates a kinetic isotope effect (KIE) in the photoreforming reaction.

As shown in Figure 5-(2) and Figure 5-(3), in the system of formic acid and formic-d acid in H_2_O, comparable amounts of H_2_ (368.4 μmol and 364.5 μmol) and CO_2_ (155.2 μmol and 160.7 μmol) were observed. In the reaction with formic-d acid, no HD and D_2_ were observed, and this could be due to either the H atom from the C–H bond in formic acid contributing insignificantly to H_2_ formation or the amount of HD formed being below the limit of detection.

In the system of formic acid in D_2_O, D_2_ (194.4 μmol) and HD (12.8 μmol) are the main hydrogen products with CO_2_ (139.3 μmol) produced. KIE was also observed when H_2_O was replaced by D_2_O in formic acid photoreforming as observed for glucose, which indicates that the hydrogen produced from the photoreforming of glucose and formic acid is mainly from the reduction of protons resulting from water oxidation.

In the photoreforming of glucose and formic acid in D_2_O, the significant reduction in hydrogen production observed was not mirrored in the CO_2_ production with comparable amounts of CO_2_ formed for reactions in H_2_O and D_2_O from glucose (14.4 μmol and 12.6 μmol) and formic acid (155.2 μmol and 139.3 μmol). The comparable CO_2_ production from the systems in H_2_O and D_2_O suggests similar substrate conversion of glucose and formic acid in both systems. Comparable conversion of the substrate while hydrogen production varied suggests oxidation of water and the substrate occur simultaneously on the catalyst and that the respective adsorption strength and surface coverage (of water vs substrate/intermediates) on the catalyst could dictate the dominant reaction pathway to produce H_2_.

### Origin of H_2_ from Photoreforming of Formic Acid: Effect of Formic Acid Concentration

Formic acid photoreforming was studied further by varying the initial concentrations (i.e., [FA] = 0.006, 6.6, and 23.9 mol L^–1^ in D_2_O). Specially, a low substrate concentration ensures a relatively high surface coverage of adsorbed water on the catalyst, and hence to investigate the role water played in the reaction pathway over Pt/m-TiO_2_.

**Figure 6:**
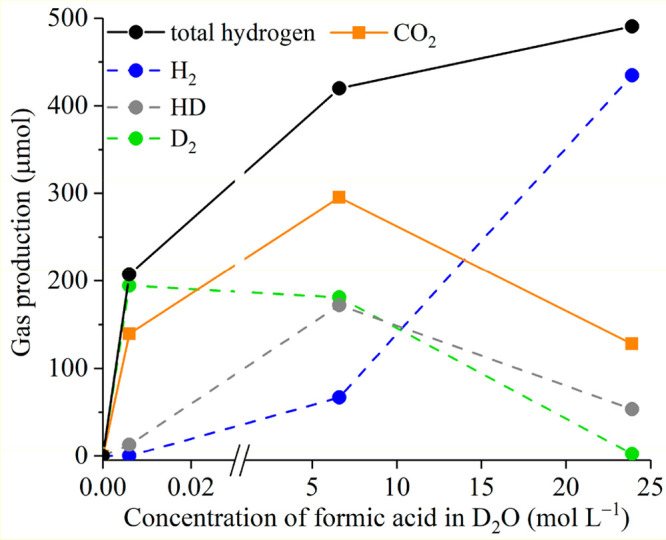
Effect of formic acid concentration in D_2_O on the composition of gas production in photoreforming over Pt/m-TiO_2_. Reaction conditions: 75 mg of Pt/m-TiO_2_, 0.006, 6.6, and 23.9 mol L^–1^ substrate in 100 mL of H_2_O, under the irradiation of UV-A lamp for 5 h at 40 °C.

**Scheme 3:**
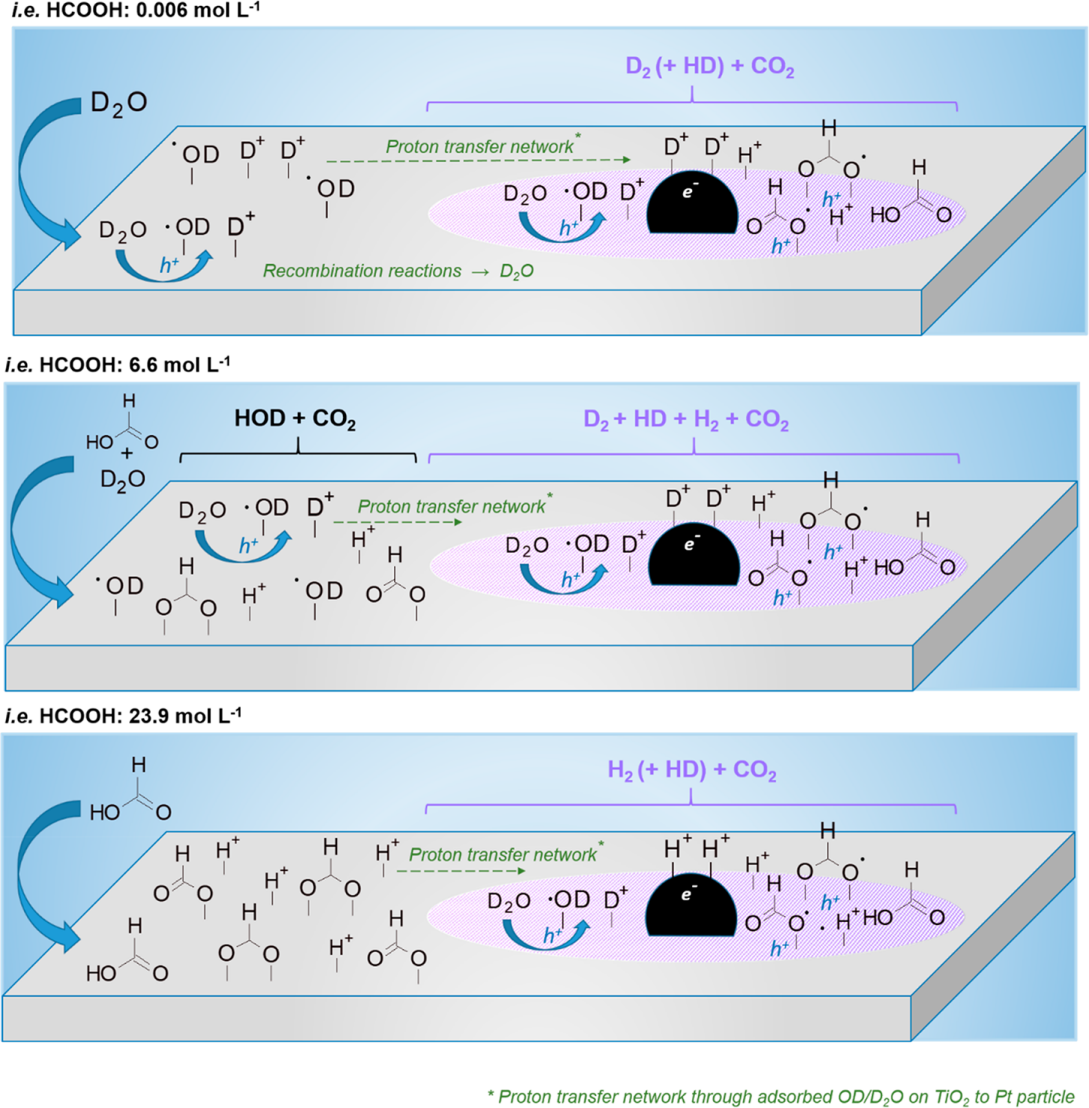
Schematic of Pt/TiO_2_ Surface during Photoreforming of Formic Acid in D_2_O at Different Substrate Concentrations

Distribution of gas products (including H_2_, HD, D_2_ and CO_2_, and total hydrogen) was shown in Figure 6. In the diluted system with [FA] = 0.006 mol L^–1^, D_2_ (194.4 μmol) was the main hydrogen product with significantly less HD formed (12.8 μmol) and no H_2_ observed. However, in the system with [FA] = 23.9 mol L^–1^, H_2_ (434.8 μmol) is the main hydrogen product with a lower amount of HD (53.4 μmol) and a trace amount of D_2_ (2.4 μmol) observed. The findings show the variation in reaction pathways for hydrogen production with the change of substrate concentration in formic acid photoreforming (Scheme 3).

In the diluted system ([FA] = 0.006 mol L^–1^), direct oxidation of water by h^+^ forming ·OD radicals and D^+^ (eq 5) could dominate due to a relatively high surface coverage of adsorbed water on the catalyst. D^+^ species undergo reduction at Pt particles by photogenerated electrons forming D_2_ (eq 6) which is the major hydrogen species formed at this formic acid concentration (Figure 6).
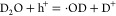
5
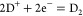
6In the concentrated system ([FA] = 23.9 mol L^–1^), direct (via h^+^) oxidation of formic acid/adsorbed formate dominates as less ·OD was formed due to the reduced concentration of D_2_O in this system and the H_2_ is derived from the protons of formic acid/formate (eqs 7–eq 10). The amount of CO_2_ produced is similar for reactions at both the high (128.0 μmol) and low (139.3 μmol) concentrations of formic acid. The CO_2_ production at the high [FA] being the same as at a significantly lower formic acid concentration suggests that (i) specific sites (proposed to be Pt-TiO_2_ interface sites) can oxidize formic acid to CO_2_ and (ii) it is the surface coverage rather than the solution concentration which controls the oxidation of formic acid to CO_2_.
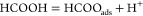
7
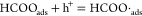
8
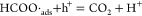
9
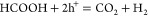
10

Interestingly, in the system with [FA] = 6.6 mol L^–1^, similar amounts of D_2_ (180.9 μmol) and HD (172.0 μmol) were observed with lower H_2_ production (66.9 μmol), indicating that the origin of protons was from both formic acid and water at this substrate concentration. Hydrogen production originating from protons derived from water and the substrate was also observed in the study of Belhadj et al. for the photoreforming of formaldehyde in aqueous solution (i.e., ∼5.4 mol L^–1^ formaldehyde over 1 g L^–1^ Pt/TiO_2_ catalyst).^66^ However, at [FA] = 6.6 mol L^–1^, a relatively higher amount of CO_2_ (295.1 μmol) was produced relative to the reaction at low (0.006 mol L^–1^) and high [FA] (23.9 mol L^–1^) while the H_2_ production was more comparable to that observed at the high formic acid concentration. At 6.6 mol L^–1^ formic acid concentration, with water and formic acid present on the surface of the catalyst an additional pathway of indirect formic acid/formate oxidation (eqs 11–13) could occur through reaction of formic acid with ·OD radicals (eq 5) forming water (HOD in D_2_O) rather than hydrogen. The additional pathway of indirect formic acid/formate oxidation would give a relative increase in the CO_2_ production relative to the H_2_ production as observed herein for photoreforming of formic acid at concentrations of 6.6 mol L^–1^ compared to 23.9 mol L^–1^.
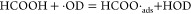
11
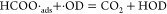
12
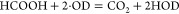
13

The isotopic studies showed that the source of the hydrogen produced in formic acid photoreforming was dependent on the relative surface coverage of adsorbed water and formic acid. In D_2_O, D_2_ was generated in the photoreforming reaction with a low concentration of formic acid via direct h^+^ oxidation of water (with formation of OD radicals), while with a high concentration of formic acid, H_2_ was the major species via direct h^+^ oxidation of formic acid. However, the production of CO_2_ from formic acid is proposed to be controlled by oxidation (direct and/or indirect pathway depending on water/formic acid ratio) occurring at the Pt-TiO_2_ interface sites.

### Proposal of the Mechanism of Glucose Photoreforming

According to previous studies,^12−14^ the first step in glucose photoreforming was the formation of an electron/hole (e^–^/h^+^) pair in the photocatalyst. The energy from photons was absorbed by the catalysts to excite e^–^ from the valence band to the conduction band, leaving h^+^ in the valence band. Then, the adsorbed water and surface hydroxyl groups could react with the photogenerated h^+^ to form adsorbed hydroxyl radicals (·OH_ads_). An *in situ* electron paramagnetic resonance (EPR) experiment has been employed as an efficient technique for probing the formation of radicals such as ·COOH, ·OH and Ph(·CH)NH_2_ in recent works.^67,68^ In this study, the formation of ·OH radicals in the system of glucose photoreforming and system with only water has been identified under the irradiation of UV-A lamp by *in situ* EPR with DMPO as the spin trapping agent (as shown in Figure S12 and S13, experiment details are shown in the ESI). Based on the analysis of photoreforming products in the liquid phase, the reaction pathway of glucose oxidation during photoreforming was proposed, as shown in Scheme 1. Conversion of glucose via oxidation (by ·OH or h^+^) was sequential via α-scission (C_1_–C_2_ bond cleavage) in the system. That is, glucose was first oxidized to arabinose and formic acid and then, arabinose was oxidized to erythrose and formic acid, followed by the conversion of erythrose to glyceraldehyde and formic acid. Conversion of glyceraldehyde could be via oxidation to glycolic acid and formic acid, or isomerization to DHA, although oxidation to glycolic acid was favored over Pt/m-TiO_2_ and isomerization of glyceraldehyde to DHA was more likely to occur over m-TiO_2_. Finally, formaldehyde and HCOOH were produced from the conversion of glycolic acid with CO_2_ produced from oxidation of formic acid, formed at each oxidation step as well as the final intermediate.

Based on the findings from the isotopic labeling experiments, H_2_ was generated mainly from water at the low concentration of glucose studied herein (0.006 mol L^–1^) through a proposed formic acid-driven mechanism, due to formic acid/formate adsorption on the surface of the catalyst at Pt/Ti interface sites.

Specifically, for reaction in D_2_O, D_2_O was first oxidized by h^+^ to form D^+^ and ·OD radicals (as shown below in eq 5). Then, D^+^ was reduced to D_2_ by e^–^ (eq 6), and the ·OD radicals could attack and degrade glucose through C_1_–C_2_ scission to release formic acid and arabinose. At a low concentration of glucose, adsorbed water on the surface of the catalyst could continuously generate H^+^/D^+^ which can be reduced to H_2_/D_2_. The ATR-IR results showed formic acid on the catalyst surface (in the presence of glucose) which could adsorb both molecularly (HCOOH_ads_) and dissociatively as formate (HCOO_ads_), with HCOOH_ads_ regarded as resupplying HCOO_ads_ (Scheme 2). Formic acid/formate can react with h^+^ (eqs 7–10) and/or ·OH (eqs 11–13), forming CO_2_ and either H_2_ (from direct h^+^ oxidation) or H_2_O (from indirect ·OH oxidation) while sustaining the charge separation for oxidation of water.

## Conclusions

Detailed comparative study of glucose photoreforming for H_2_ production over m-TiO_2_ and Pt/m-TiO_2_ was carried out via products analysis (i.e., by GC, HPLC, and isotope MS) with the aid of in situ ATR-IR characterization to probe the species adsorbed on the catalyst under photoreforming conditions. The Pt loading on m-TiO_2_ improved H_2_ production significantly in glucose photoreforming, i.e., by 13.4 times compared to that over the pristine m-TiO_2_. High selectivity to formic acid and arabinose suggests the α-scission (or C_1_–C_2_ bond cleavage) mechanism of glucose photoreforming over both m-TiO_2_ and Pt/m-TiO_2_ systems. In comparison to the m-TiO_2_ system, the Pt/m-TiO_2_ system showed lower selectivity to formic acid, which could be attributed to formic acid adsorption/conversion on the surface of Pt/m-TiO_2_. *In situ* ATR-IR characterization suggested that formic acid could be adsorbed and converted continually over Pt/m-TiO_2_ under UV irradiation, while it was not converted over m-TiO_2_. Based on the results from isotopic-MS studies, at low glucose concentration, the protons in water contributed predominantly to H_2_ generation.

In this study, detailed product analysis combined with the *in situ* characterization of the adsorbed species formed during photoreforming process over the catalysts provides a better understanding of the reaction mechanism of glucose photoreforming. The developed method can be a valuable tool in mechanistic investigation of photocatalytic systems.

## Data Availability

The open access data is available on The University of Manchester - Institutional Repository (https://www.research.manchester.ac.uk/portal).

## Supporting Information Available

Design of ATR-IR cell; Information of UV LED; Liquid products in the photoreforming of glucose and its intermediates based on HPLC analysis; ATR-IR spectra of glucose and formic acid over catalysts; H_2_ production of photoreforming of glucose and its intermediates; H_2_ production from control experiments; Time course of gas products for isotopic experiments; *In situ* EPR experiments under UV irradiation (PDF)
