# Supplementary material for: Mechanistic Study of Glucose Photoreforming over TiO2-Based Catalysts for H2 Production
Source: ACS Catal. 2023 Jun 13;13(13):8574–87. doi: 10.1021/acscatal.3c00858 (PMC10334428; doi:10.1021/acscatal.3c00858)
Supplement: Supplementary file 1 — cs3c00858_si_001.pdf [file cs3c00858_si_001.pdf]

Electronic Supporting Information

Mechanistic study of glucose photoreforming over TiO_2-based catalysts for H_2 production

Lan Lan[a*], Helen Daly[a*], Rehana Sung[b], Floriana Tuna[cd], Nathan Skillen[e], Peter K. J. Robertson[e] , Christopher Hardacre[a,*], Xiaolei Fan[a]

[a] *Department of Chemical Engineering, School of Engineering, The University of Manchester, M13 9PL, United Kingdom*

[b] *Manchester Institute of Biotechnology, The University of Manchester, M13 9PL, United Kingdom*

[c] *Department of Chemistry, University of Manchester, Manchester, M13 9PL, United Kingdom*

[d] *Photon Science Institute, University of Manchester, Manchester, M13 9PL, United Kingdom*

[c] *School of Chemistry and Chemical Engineering, Queen's University Belfast, BT9 5AG, United Kingdom*

[*] Corresponding authors. E-mail addresses: c.hardacre@manchester.ac.uk (C.H.); lan.lan@manchester.ac.uk (L.L.); helen.daly@manchester.ac.uk (H.D.)

Contents

1-Figure S1 The design of ATR-IR cell for in-situ characterisation of photoreforming under UV radiation

2-The information of UV LED used in the ATR-IR experiment with the spectral output of the LED shown in Figure S2

3-Figure S3 The selectivity of liquid products in glucose photoreforming as a function of irradiation time

4-Figure S4 Product selectivity as a function of radiation time in photoreforming of (a) arabinose, (b) erythrose, (c) glyceraldehyde and (d) glycolic acidover (1) m-TiO_2 and (2) Pt/m-TiO_2

5-Figure S5 HPLC analysis of product distribution in glycolic acid photoreforming over (a) m-TiO_2 and (b) Pt/m-TiO_2

6-Figure S6. ATR-IR spectra for dark adsorption of (a) glucose over a blank Ge crystal (blue), Pt/m-TiO_2 catalyst layer (black) and m-TiO_2 catalyst layer (red), and (b) arabinose over Pt/TiO_2

7- Figure S7 ATR-IR spectra of 0.1 M glucose in water over (a) m-TiO_2 and (b) 0.16%Pt/m-TiO_2 under UV irradiation (LED emission at 391 nm) for 30 min. Spectra shown are difference spectra where initial spectrum of 0.1 M glucose in water in the dark has been subtracted from all spectra recorded under different irradiation times. Bands labelled red are due to molecularly adsorbed formic acid, black to formates and blue to arabinose/loss of glucose.

8-Figure S8 ATR-IR spectra under UV irradiation, 0.1 M glucose (black spectrum) and 0.1 M formic acid (red spectrum) over Pt/TiO_2

9-Figure S9 H_2 production of photoreforming of glucose and its intermediates over (1) m-TiO_2 and (2) Pt/TiO_2

10-Figure S10 H_2 production from control experiments of the catalytic decomposition of formic acid without light irradiation (black line), and the photo degradation of formic acid without catalyst (blue line), reaction conditions: 75 mg of Pt/m-TiO_2, 100 g formic acid in 100 mL H_2O/D_2O, under irradiation of the UV-A lamp for 3 h at 40 °C

11-Figure S11 Time course of gas production (H_2, HD and D_2) over Pt/m-TiO_2 in (a) photoreforming of glucose in H_2O/D_2O, and (b) photoreforming of formic acid or formic-d acid in H_2O/D_2O, reaction conditions: 75 mg of Pt/m-TiO_2, 0.006 mol L−1 substrate in 100 mL H_2O/D_2O, under the irradiation of UV-A lamp for 5 h at 40 °C

12-*In-situ* EPR experiment under UV irradiation. The *in-situ* EPR spectra of pure DMPO in water, glucose photoreforming and system without glucose were shown in the Figure S12 and Figure S13.

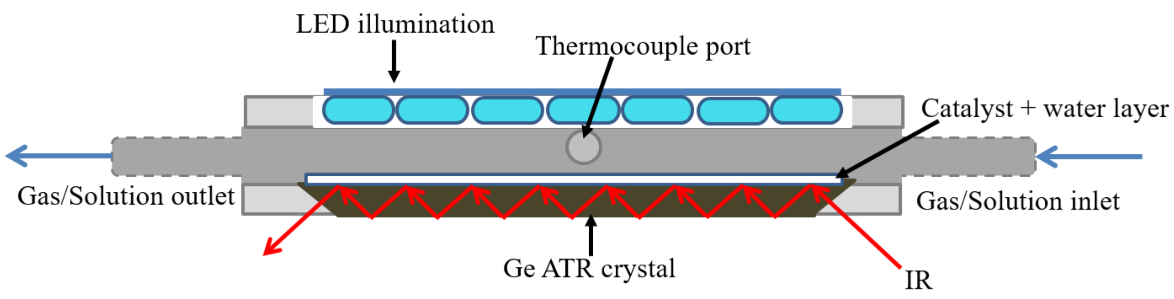

Figure S1. ATR-IR cell with the Ge ATR crystal for in-situ characterisation of photoreforming under UV radiation

UV LED information

Illumination of UV LED in the ATR experiment was provided by a small array of 7 UV-LEDs (UV5TZ, Bivar) mounted linearly onto printed circuit board (RS Components, UK) and positioned directly over the ATR flow cell. The array was 90 mm in length and consisted of 2-pin 5 mm UV-LEDs with a peak wavelength of 390 nm, viewing angle of 30 ° and were operated at V_F = 3.2 dcV and I_F = 0.038 A (0.12 W) *via* a dedicated bench top DC power supply (ADD SUPPLIER DETAILS HERE). Figure S2 provides a spectral output of the lamp as measured by a StellarNet BLACK-comet concave grating spectrometer (StellarNet Inc., USA).

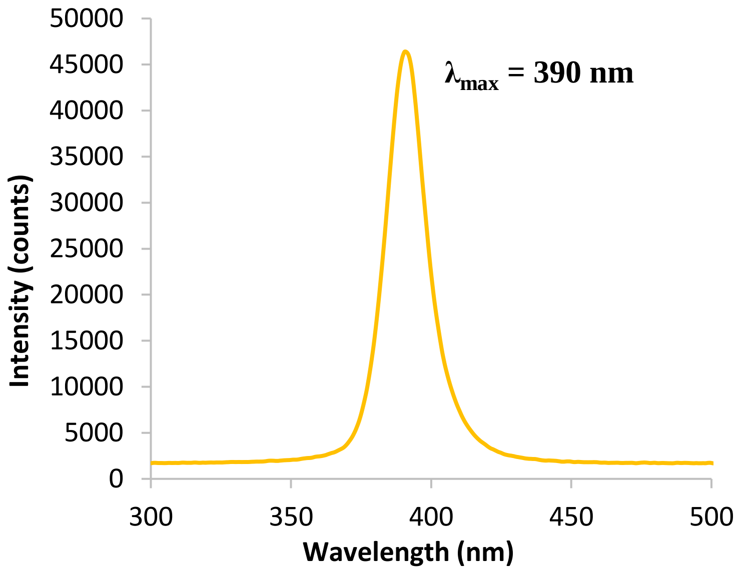

Figure S2. A spectral output of the UV LED used in the ATR experiment

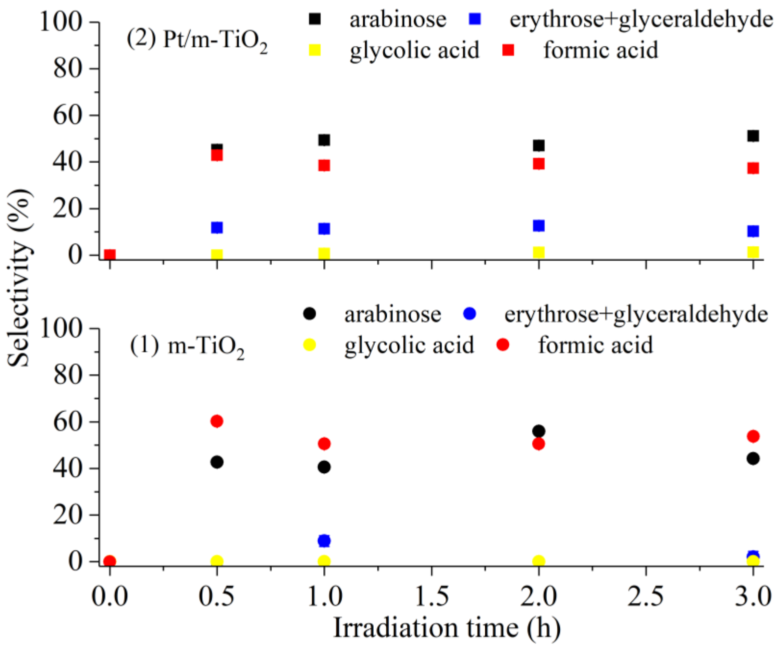

Figure S3. Selectivity to liquid-phase products from glucose photoreforming as a function of radiation time over (1) m-TiO_2 and (2) Pt/m-TiO_2, legends: arabinose (black), glycolic acid (yellow), erythrose+glyceraldehyde (blue) and formic acid (red).

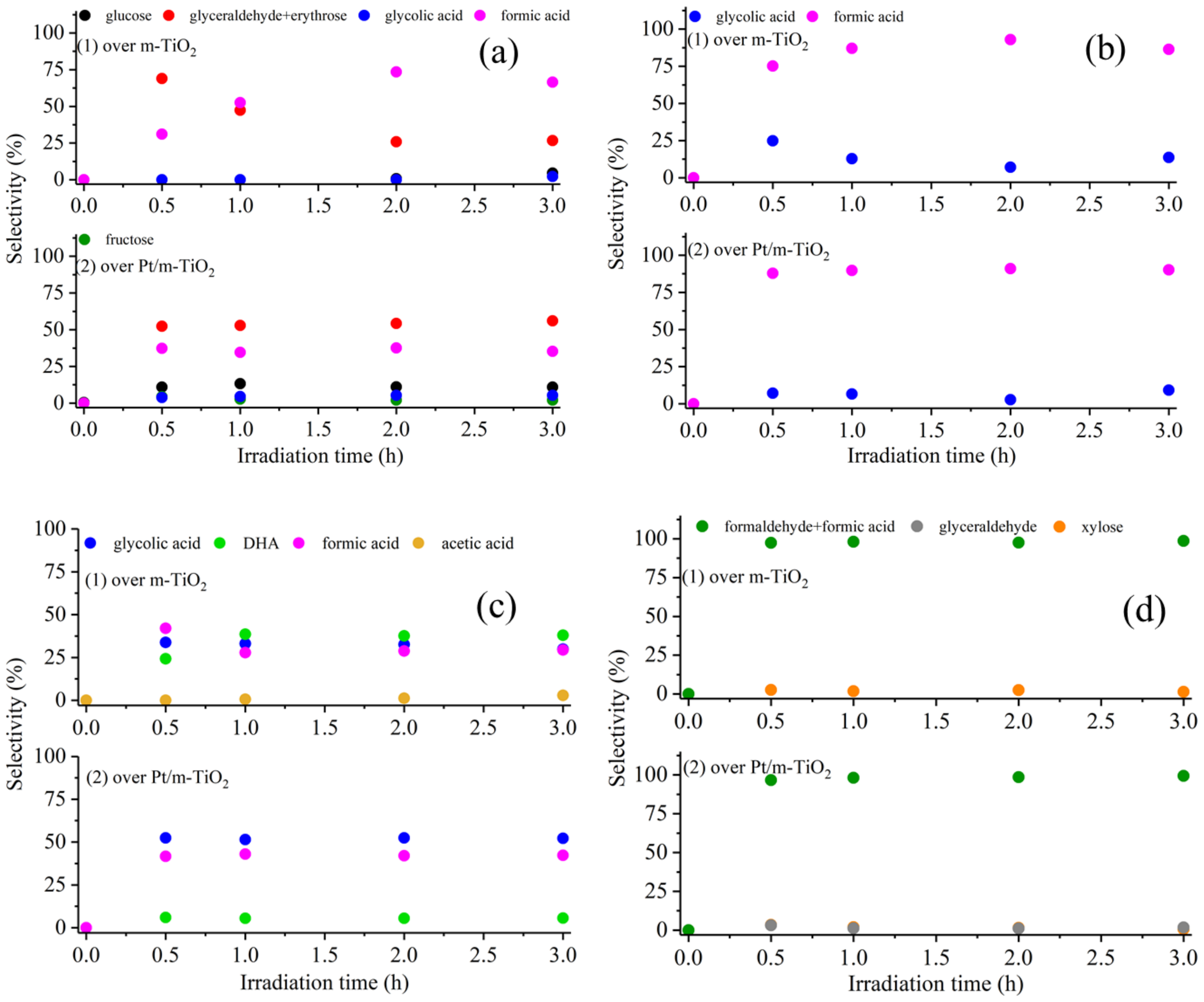

Figure S4. Product selectivity as a function of radiation time in photoreforming of (a) arabinose, (b) erythrose, (c) glyceraldehyde and (d) glycolic acidover (1) m-TiO_2 and (2) Pt/m-TiO_2. Reaction conditions: 75 mg of Pt/m-TiO_2, 100 g of substrate in 100 mL H_2O, under UV radiation for 3 h at 40 °C.

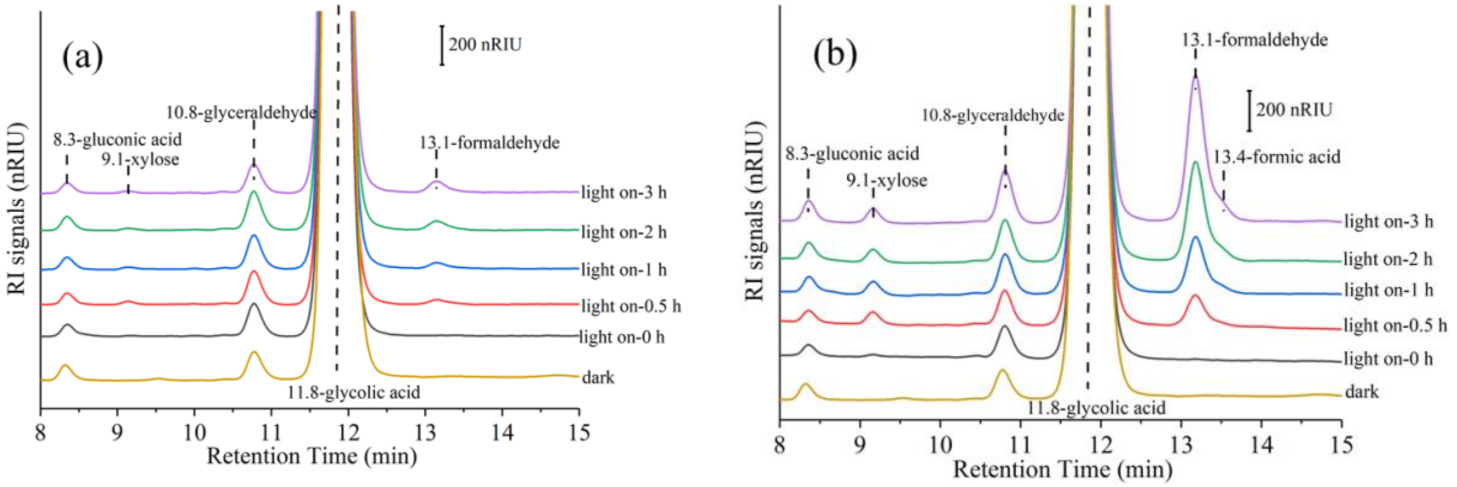

Figure S5. HPLC analysis of product distribution in glycolic acid photoreforming over (a) m-TiO_2 and (b) Pt/m-TiO_2

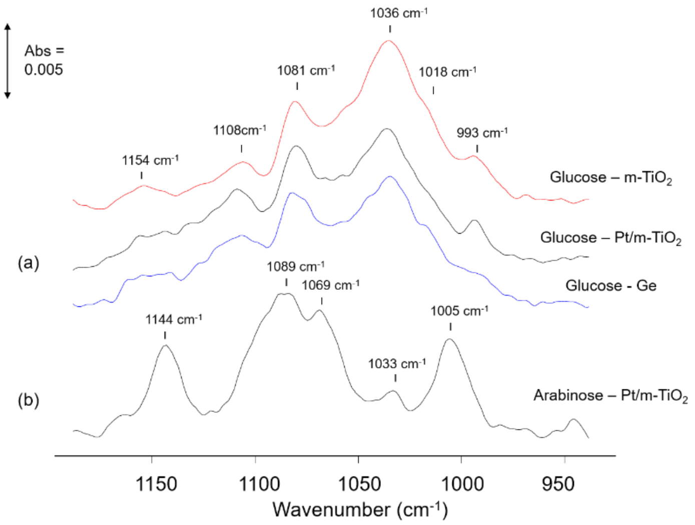

Figure S6. ATR-IR spectra for dark adsorption of (a) glucose over a blank Ge crystal (blue), Pt/m-TiO_2 catalyst layer (black) and m-TiO_2 catalyst layer (red), and (b) arabinose over Pt/TiO_2

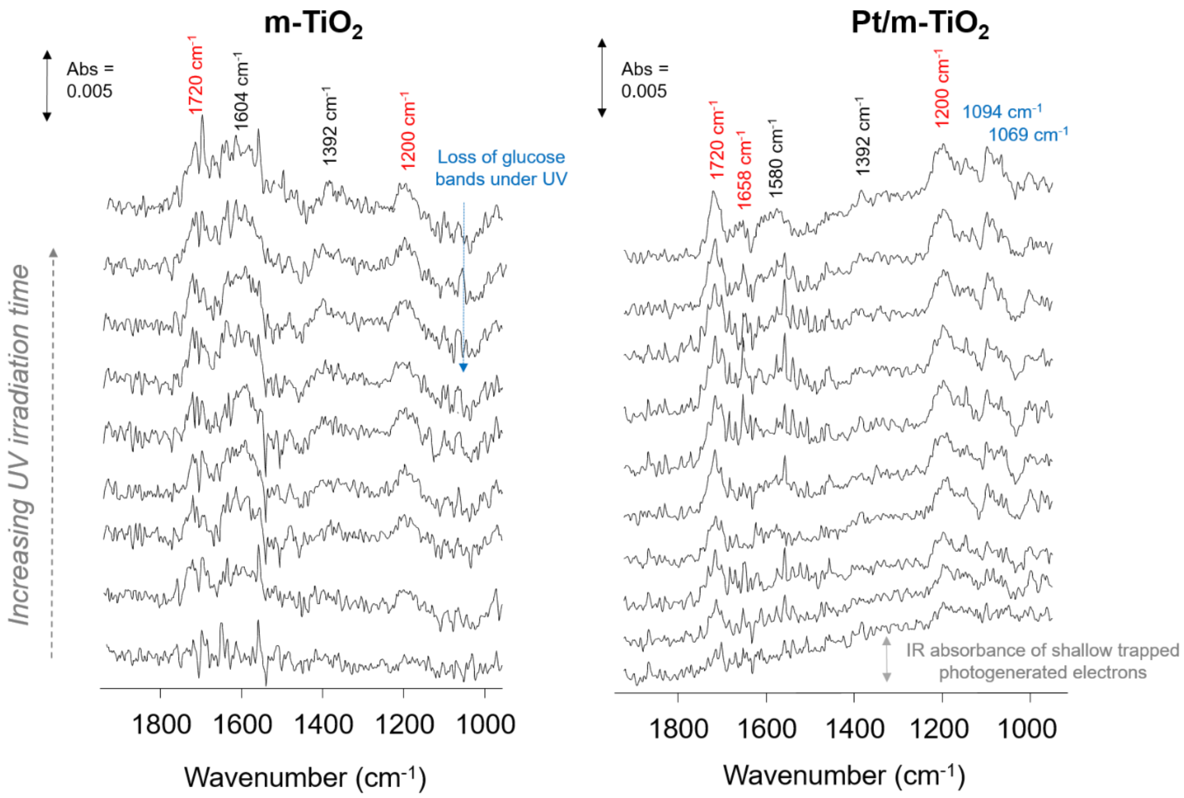

Figure S7. ATR-IR spectra of 0.1 M glucose in water over (a) m-TiO_2 and (b) 0.16%Pt/m-TiO_2 under UV irradiation (LED emission at 391 nm) for 30 min. Spectra shown are difference spectra where initial spectrum of 0.1 M glucose in water in the dark has been subtracted from all spectra recorded under different irradiation times. Bands labelled red are due to molecularly adsorbed formic acid, black to formates and blue to arabinose/loss of glucose.

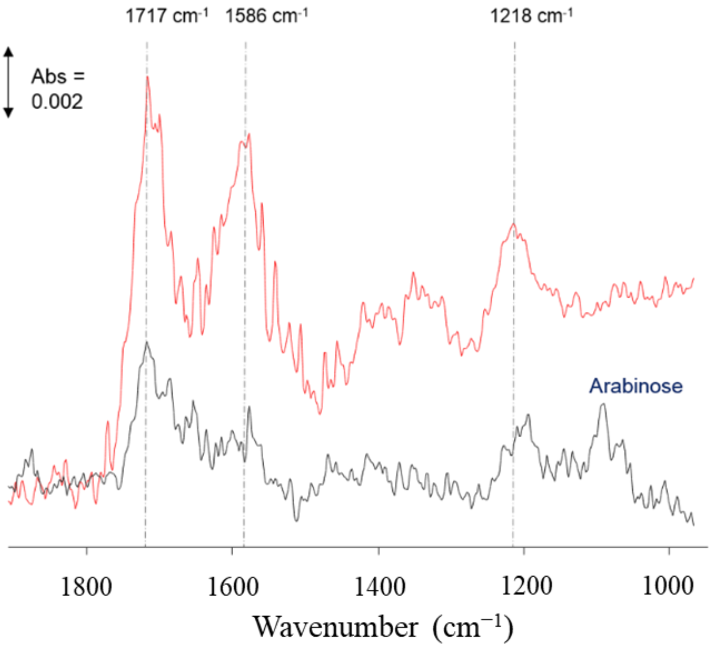

Figure S8. ATR-IR spectra under UV irradiation, 0.1 M glucose (black spectrum) and 0.1 M formic acid (red spectrum) over Pt/m-TiO_2

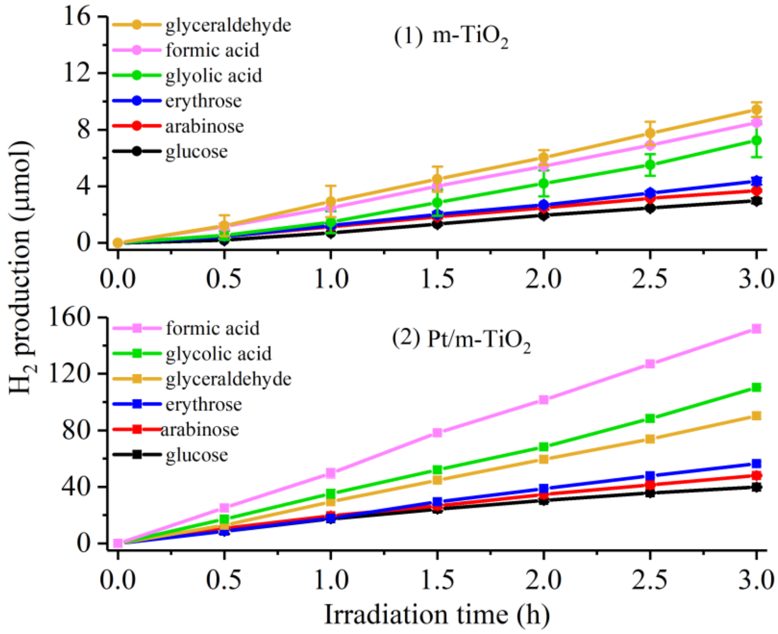

Figure S9. H_2 production of photoreforming of glucose and its intermediates over (1) m-TiO_2 and (2) Pt/TiO_2

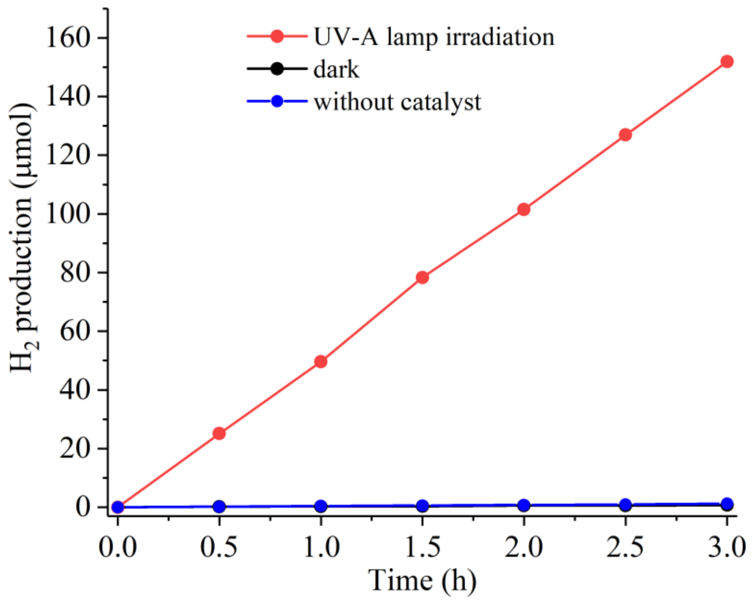

Figure S10. **H_2 production from control experiments of the catalytic decomposition of formic acid without light irradiation (black line), and the photo degradation of formic acid without catalyst (blue line), reaction conditions: 75 mg of Pt/m-TiO_2, 100 g formic acid in 100 mL H_2O/D_2O, under irradiation of the UV-A lamp for 3 h at 40 °C**

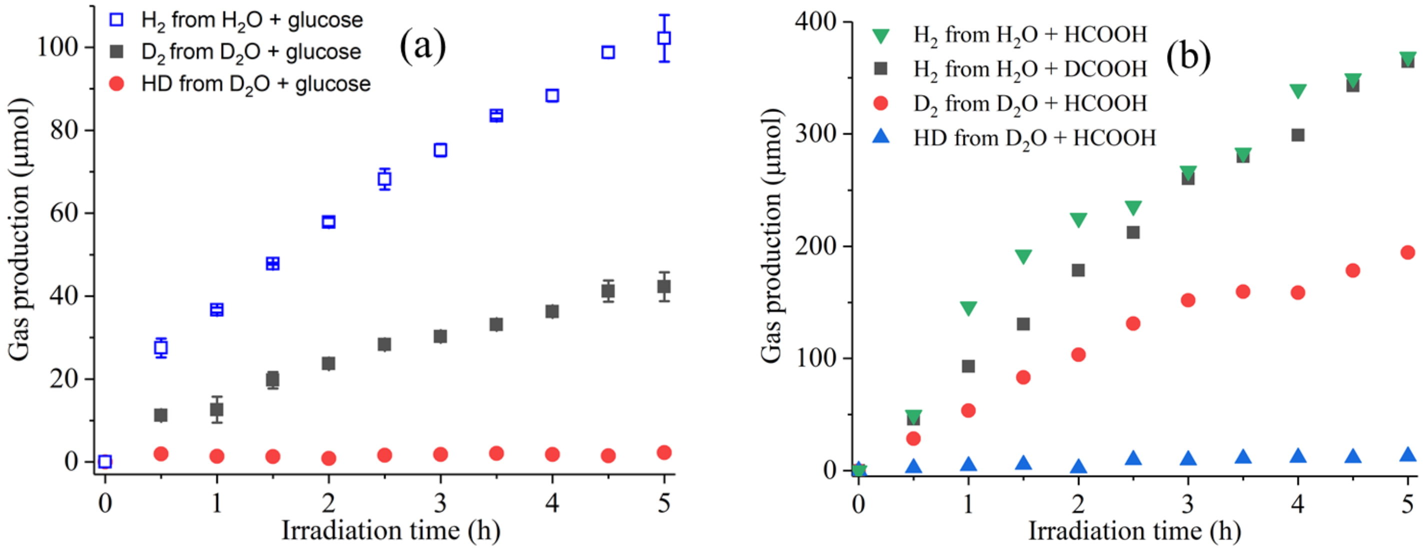

Figure S11. Time course of gas production (H_2, HD and D_2) over Pt/m-TiO_2 in (a) photoreforming of glucose in H_2O/D_2O, and (b) photoreforming of formic acid or formic-d acid in H_2O/D_2O, reaction conditions: 75 mg of Pt/m-TiO_2, 0.006 mol L^{-1} substrate in 100 mL H_2O/D_2O, under the irradiation of UV-A lamp for 5 h at 40 °C

***In-situ* electron paramagnetic resonance (EPR) under UV irradiation**

EPR spectra was collected at X-band (ca. 9.87 GHz) on a Bruker EMX Micro EPR spectrometer at room temperature with microwave power of 2 mW and a modulation amplitude of 1 G. 5,5-dimethyl-1-pyrroline N-oxide (DMPO) was dissolved in Ar-degassed (at 80 ml min^{-1} for 30 min) DI water and used as a spin trap; a Bruker strong pitch (g = 2.0028) reference was used as a calibrator. Theoretical modelling of the spectra was performed with the EasySpin toolbox within Matlab [1]. In a typical *in-situ* EPR experiment, Pt/m-TiO_2 catalyst (packing height of 3 mm in the EPR tube) was mixed with 0.5 mL DMPO solution (4 mmol L^{-1}) in a quartz EPR tube (inner diameter: 1 mm, wall thickness: 1mm). Glucose was dissolved in the DMPO (1 g L^{-1} glucose in DMPO solution) for identifying the formation of radicals in glucose photoreforming by *in-situ* EPR. The *in-situ* EPR spectra were measured before and under the irradiation of UV-A lamp (the same one used in the typical photoreforming experiment) to probe the formation of radicals in both glucose photoreforming and system without glucose.

The in-situ EPR spectra of pure DMPO, glucose photoreforming and system without glucose were shown in the Figure S12 and Figure S13. The typical DMPO-OH signals (in water) [2] could be only observed under the irradiation of UV-A lamp in photoreforming of glucose (Figure S12-(c)) and water splitting (Figure S12-(d)), which confirmed the formation of ·OH radicals in the system of glucose photoreforming and system without glucose under the UV irradiation. The DMPO-OH signals were identified by matching perfectly with the theoretical simulated DMPO-OH signals (g = 2.0054, $A(^{1}H) = A(^{14}N)$ = 41.8 MHz (14.8 G) as shown in Figure S13.

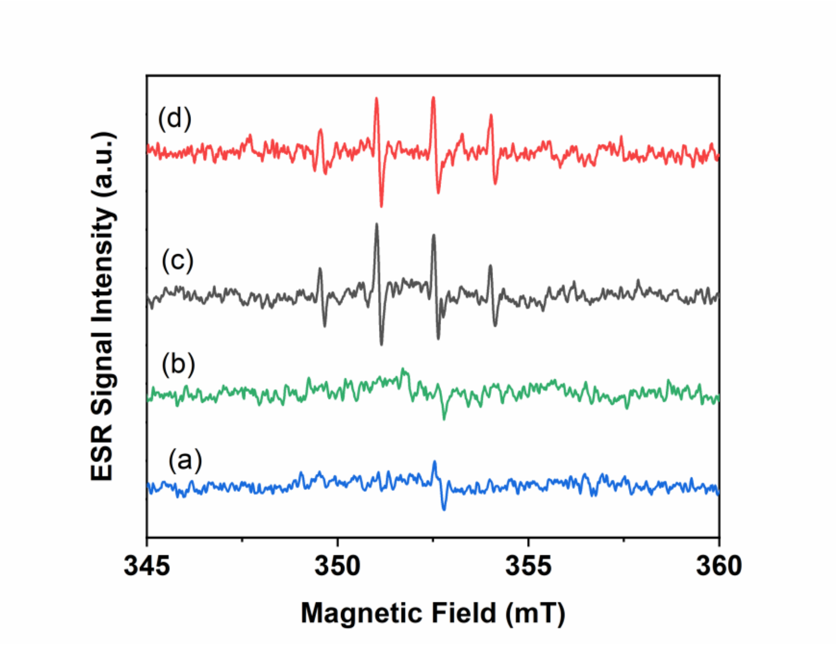

Figure S12. X-band (9.875 GHz) EPR spectra at room temperature of (a) DMPO spin trap in H_2O irradiated at 365 nm (UV-A); (b) system of Pt/m-TiO_2, glucose, and DMPO in H_2O before UV irradiation; (C) same as (B) but under the irradiation; (D) system of Pt/m-TiO_2 in H_2O only with DMPO irradiated at 365 nm, measured with MA of 1 G, microwave power of 2 mW, showing the formation of ·OH radicals via irradiation.

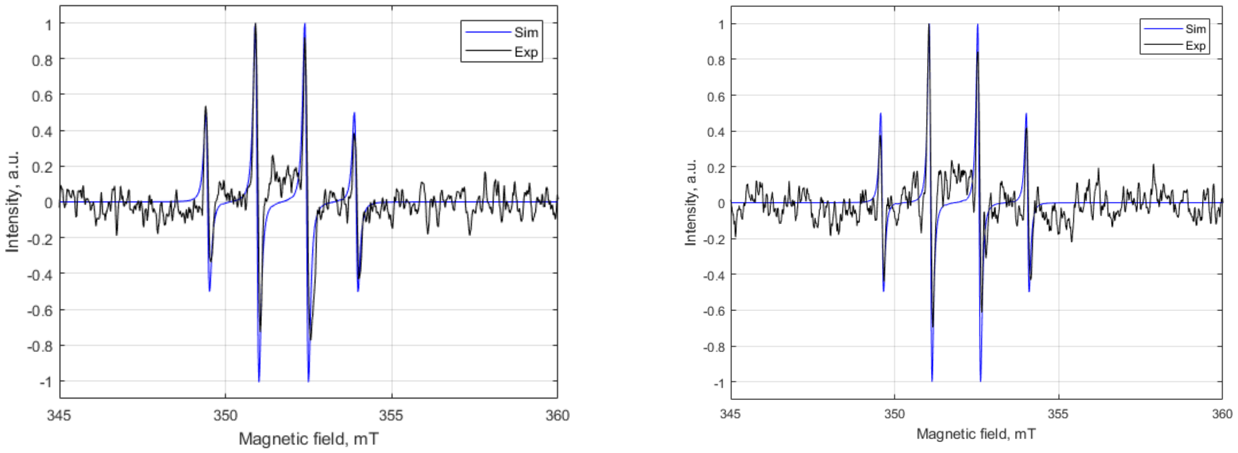

Figure S13. (a) Spin trapping EPR experiments using DMPO at X-band (9.872 GHz) for Pt/TiO_2 in H_2O irradiated with UV light of 365 nm (black), showing DMPO-OH, and its simulation (blue) with parameters g = 2.0054, A(^{1}H) = A(^{14}N) = 41.8 MHz (14.8 G). (b) Spin trapping experiments using DMPO at X-band (9.875 GHz) for Pt/TiO_2 and glucose in H_2O irradiated with UV light of 365 nm (black), showing DMPO-OH, and its simulation (blue) with parameters g = 2.0054, A(^{1}H) = A(^{14}N) = 41.8 MHz (14.8 G).

References

1. Modern EPR spectroscopy: beyond the EPR spectrum. *Phys Chem Chem Phys* **2009,** *11* (31), 6553-4.

2. Buettner, G. R., Spin Trapping: ESR parameters of spin adducts 1474 1528V. *Free Radical Biol. Med.* **1987,** *3* (4), 259-303.
